# Expression of Concern: Early intervention with ColdZyme mouth spray after self-diagnosis of common cold: A randomized, double-blind, placebo-controlled study

**DOI:** 10.1371/journal.pone.0351708

**Published:** 2026-06-16

**Authors:** The PLOS One Editors

Following the publication of this article [1], concerns were raised that the results and conclusions were reported in a manner that was misleading as to the trial’s key results.

*PLOS One* consulted two members of the Editorial Board, who advised that the data seem to support most of the article’s findings, but the reporting does not adequately balance the discussion of results, which did not indicate a statistically significant effect, and does not sufficiently discuss the study’s limitations. The article also includes sentences which are not supported, or which are erroneous or otherwise misleading as to the trial’s outcomes.

The specific concerns include:

- The results for the trial’s primary (“impact on quality of life during a common cold episode”, assessed over an 8 day period) and secondary (symptom severity during an 8 day period, exposure to concomitant treatment that may affect cold symptoms) endpoints did not indicate statistically significant results between ColdZyme and control treatments. The non-significant results are reported in the article but are not adequately reflected in the article’s conclusions.
- The *PLOS One* Editors are concerned about the reliability of the article’s claims about impacts of ColdZyme on cold duration and recovery time. These claims are not supported by data for the secondary endpoints specified in the study protocol (number of days from start of treatment until Jackson score <5, and percentage of subjects with confirmed cold symptoms at Visit 2). Instead, these claims rely on results of exploratory analyses, namely the number of days with WURSS-21 QoL > 0 and number of days of Jackson symptom score >0. For the latter, there was not a statistically significant difference between treatment and placebo groups (p = 0.087) but the article reports these data as “indicating the median time to recovery was 1 day sooner for the ColdZyme group”.
- The *PLOS One* Editors are concerned that several of the reported exploratory outcomes focus on a single day in the time course (day 5) when results favored the hypothesis. For one of the day 5 exploratory outcomes, the results statement reports a difference in symptom severity although there was not a statistically significant difference between treatment groups. “The assessment of symptom severity per Jackson score showed 11% less severe symptoms in the ColdZyme group (4.8 ± 5.0) compared to placebo (5.4 ± 4.8, p = 0.066).”
- The article uses language, e.g., “trends for significance” and “beneficial effect” that implies positive findings of Coldzyme’s efficacy rather than objectively stating the results and noting which are vs. are not statistically significant. There is also an emphasis on exploratory outcomes that are favorable to Coldzyme, including some that did not reach statistical significance.
- In addition, contrary to the Data Availability statement in [1], the individual-level underlying data supporting the article’s results were not provided. The datasets supporting Table 1 and the other reported results in [1] are now included with this notice as S1–S3 Data.

The *PLOS One* Editors issue this Expression of Concern to inform readers of the above issues. The article’s results and conclusions should be interpreted accordingly.

The authors stated that the article describes an unsuccessful trial, and that the analysis results for which concerns were raised were not included in the formal evaluation of a pre-specified hypothesis, but rather were exploratory, focusing on patterns, relationships, or unexpected discoveries that could lead to the development of new hypotheses to be investigated in new studies.

## Supporting information

S1 Data Data dictionary and variable mapping for individual-level raw data. (PDF)

S2 Data Individual-level dataset. (XLSX)

S3 Data TableC4_39_minimal_dataset. (XLSX)
