# Supplementary material for: Early intervention with ColdZyme mouth spray after self-diagnosis of common cold: A randomized, double-blind, placebo-controlled study
Source: PLoS One. 2023 Jan 18;18(1):e0279204. doi: 10.1371/journal.pone.0279204 (PMC9847898; doi:10.1371/journal.pone.0279204)

**PONE-D-22-17282**

**Data in manuscript**

- Data reported in row 266. Reference: Table A.2.2


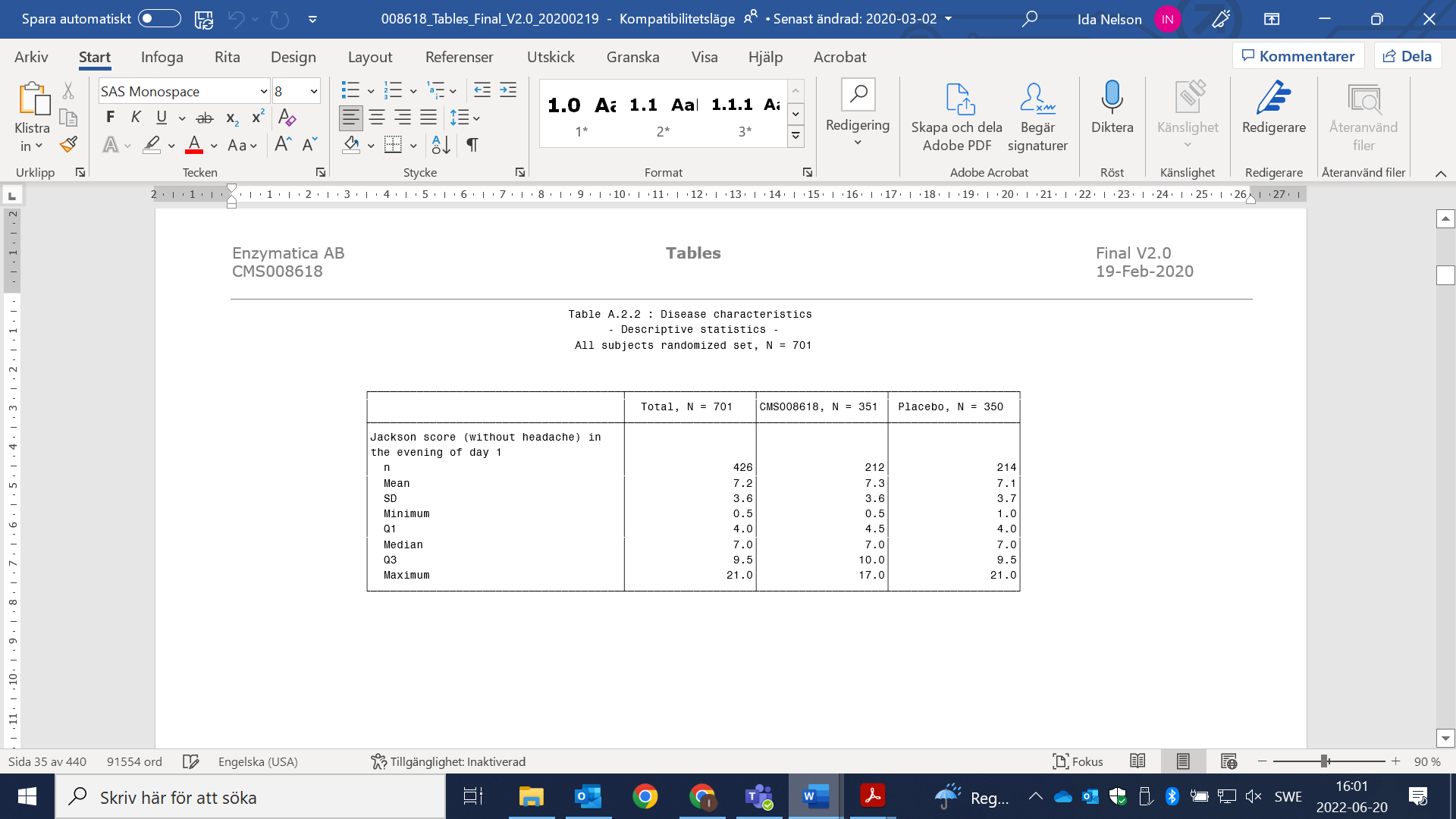


- Data reported in row 275. Reference: Table C.4.1


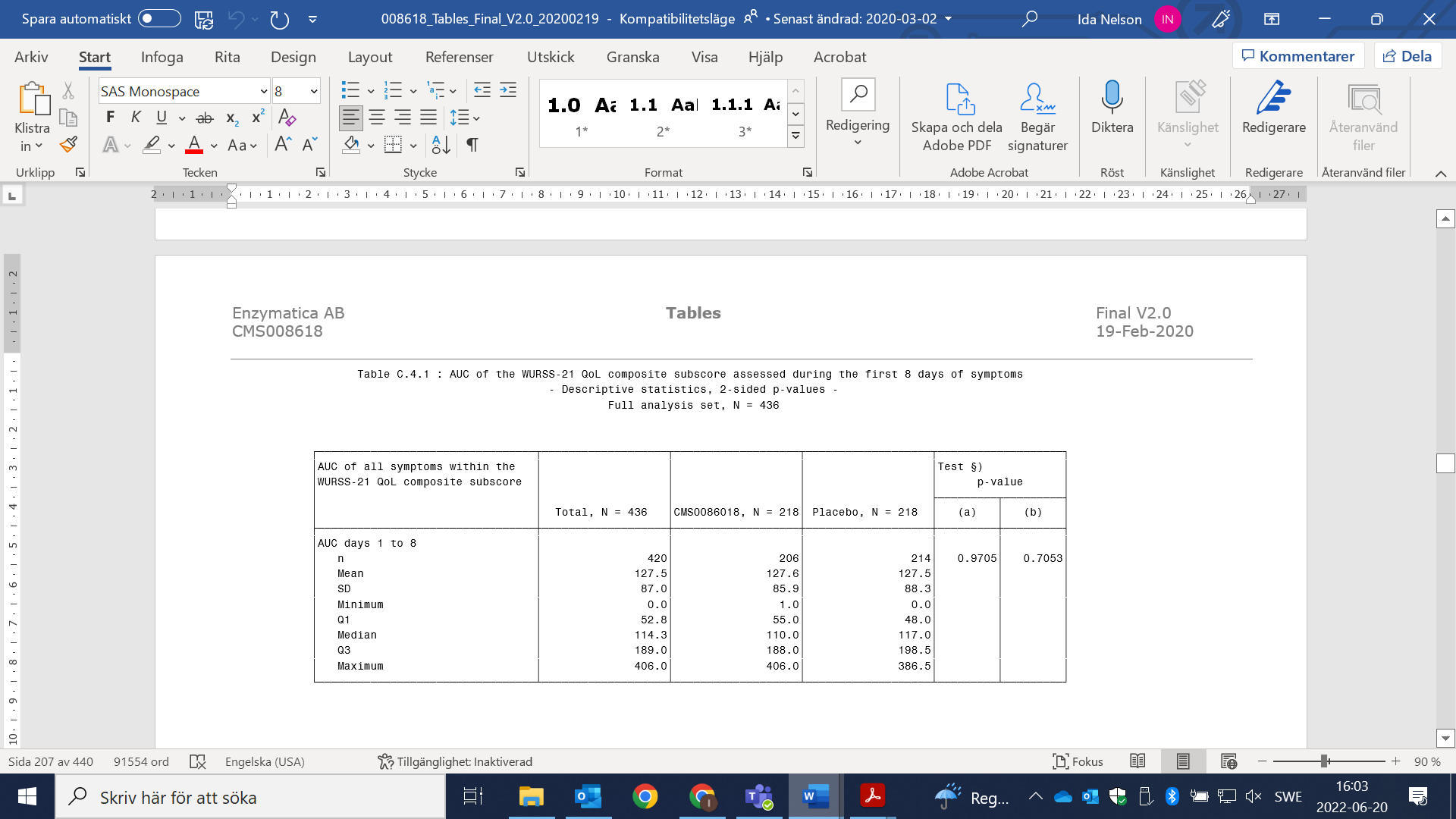


- Data reported in row 282. Reference: Table C.4.3


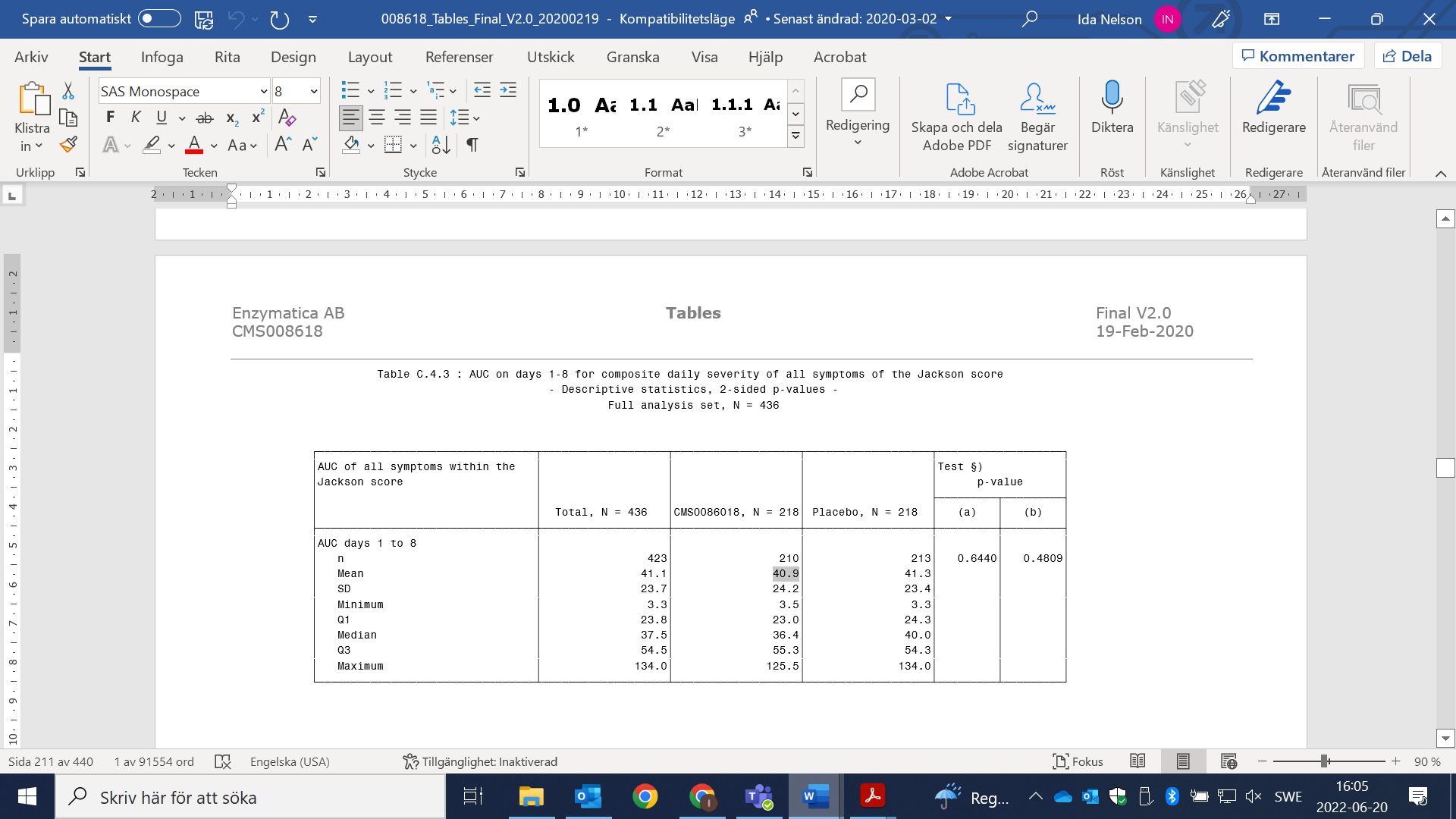


- Data reported in row 288. Reference: Table C.4.5.1


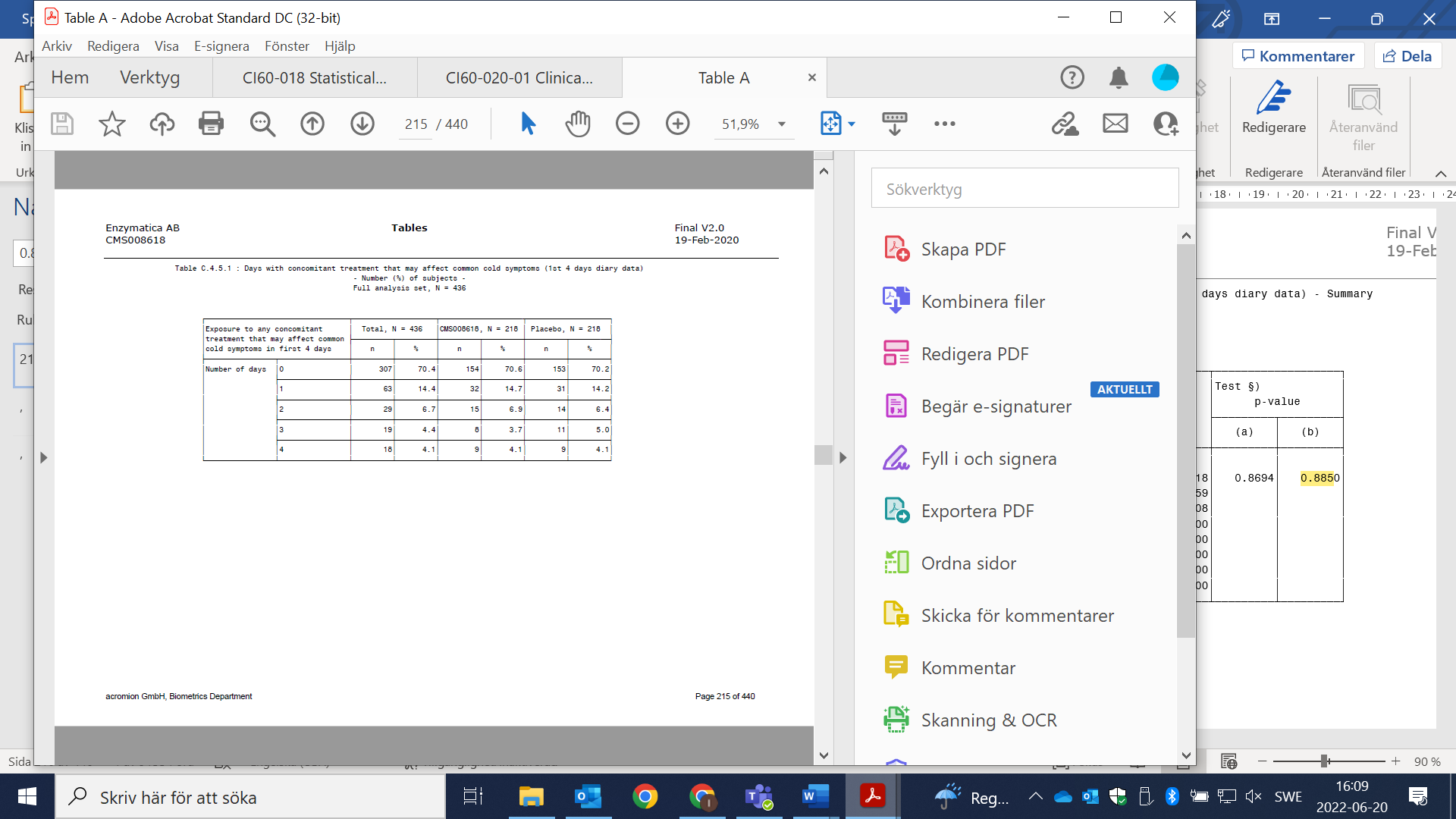


- Data reported in row 295. Reference: Table C.4.9


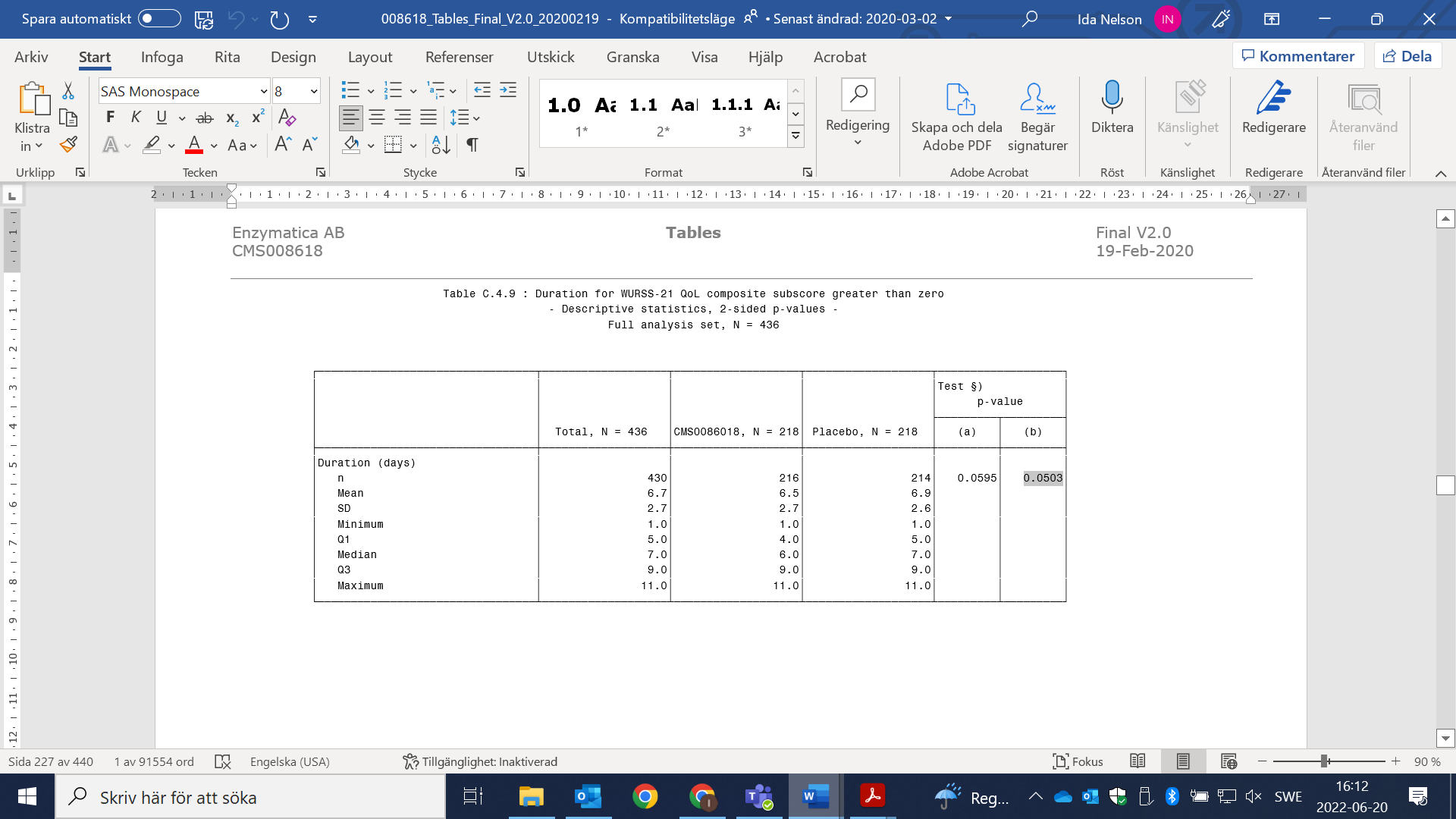


- Data reported in row 298. Reference: Table C.4.20


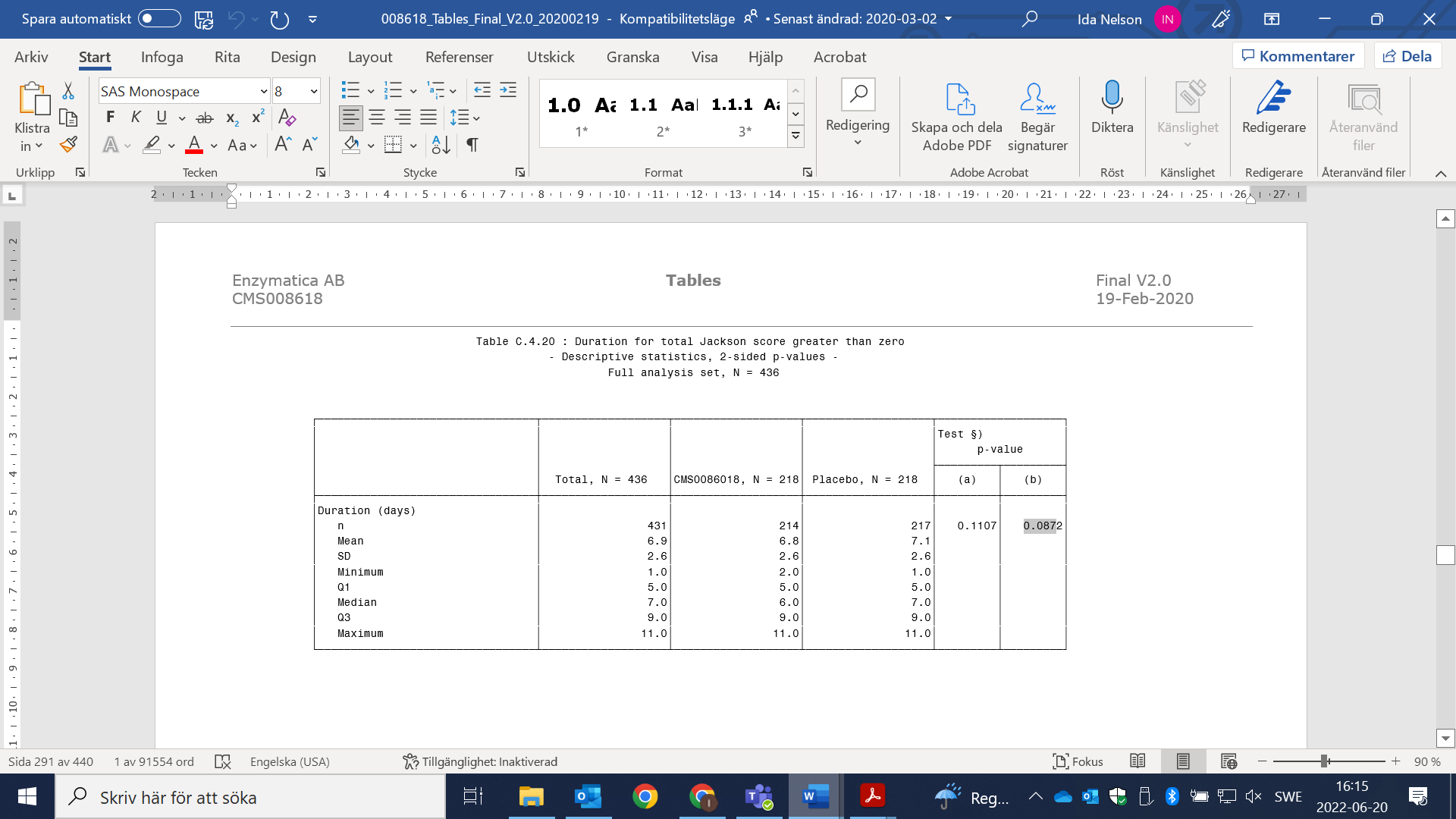


- Data reported in row 305. Reference: Table C.4.19


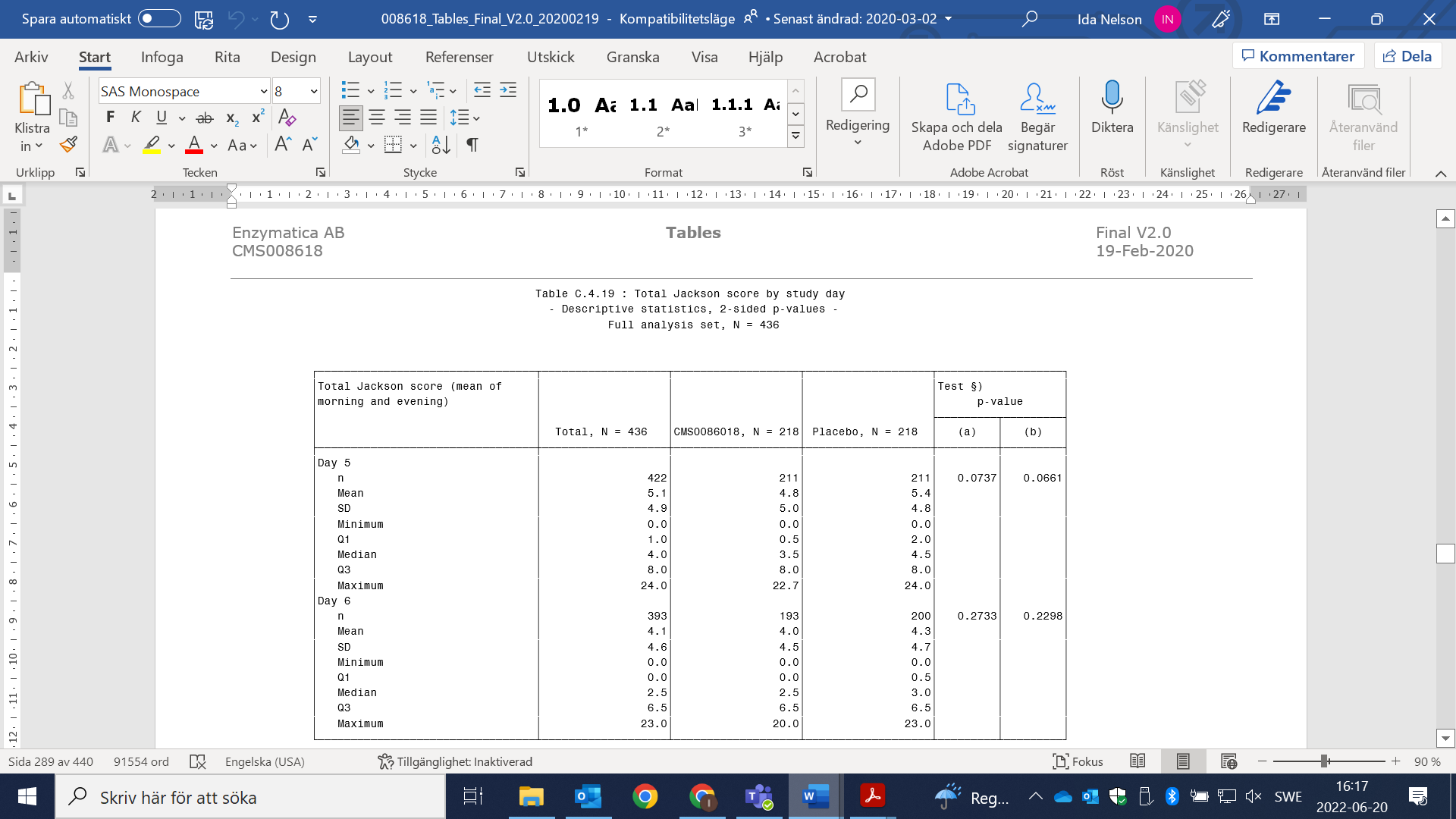


- Data reported in row 307. Reference: Table C.4.12


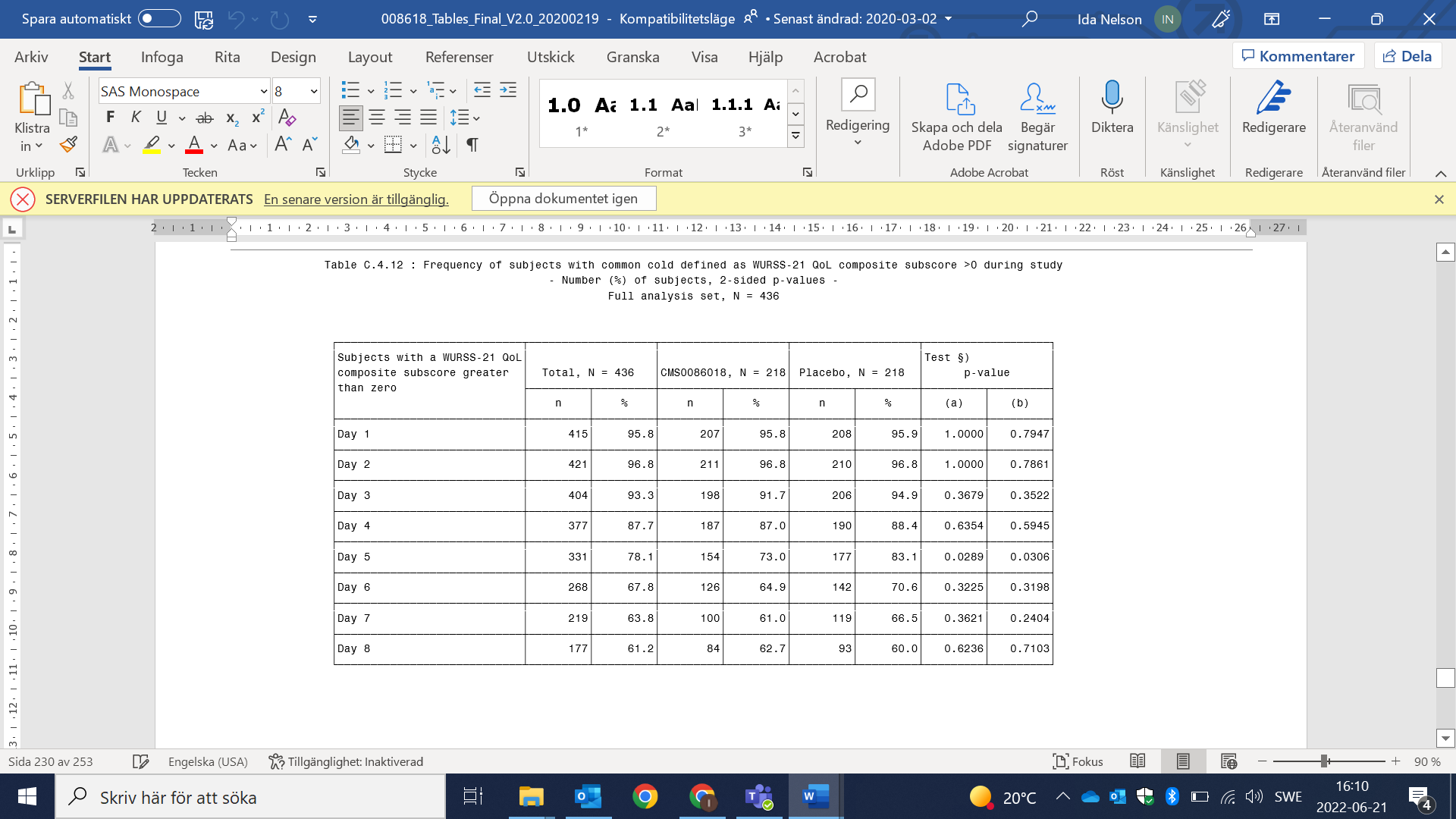


- Data reported in row 309. Reference: Table C.4.39


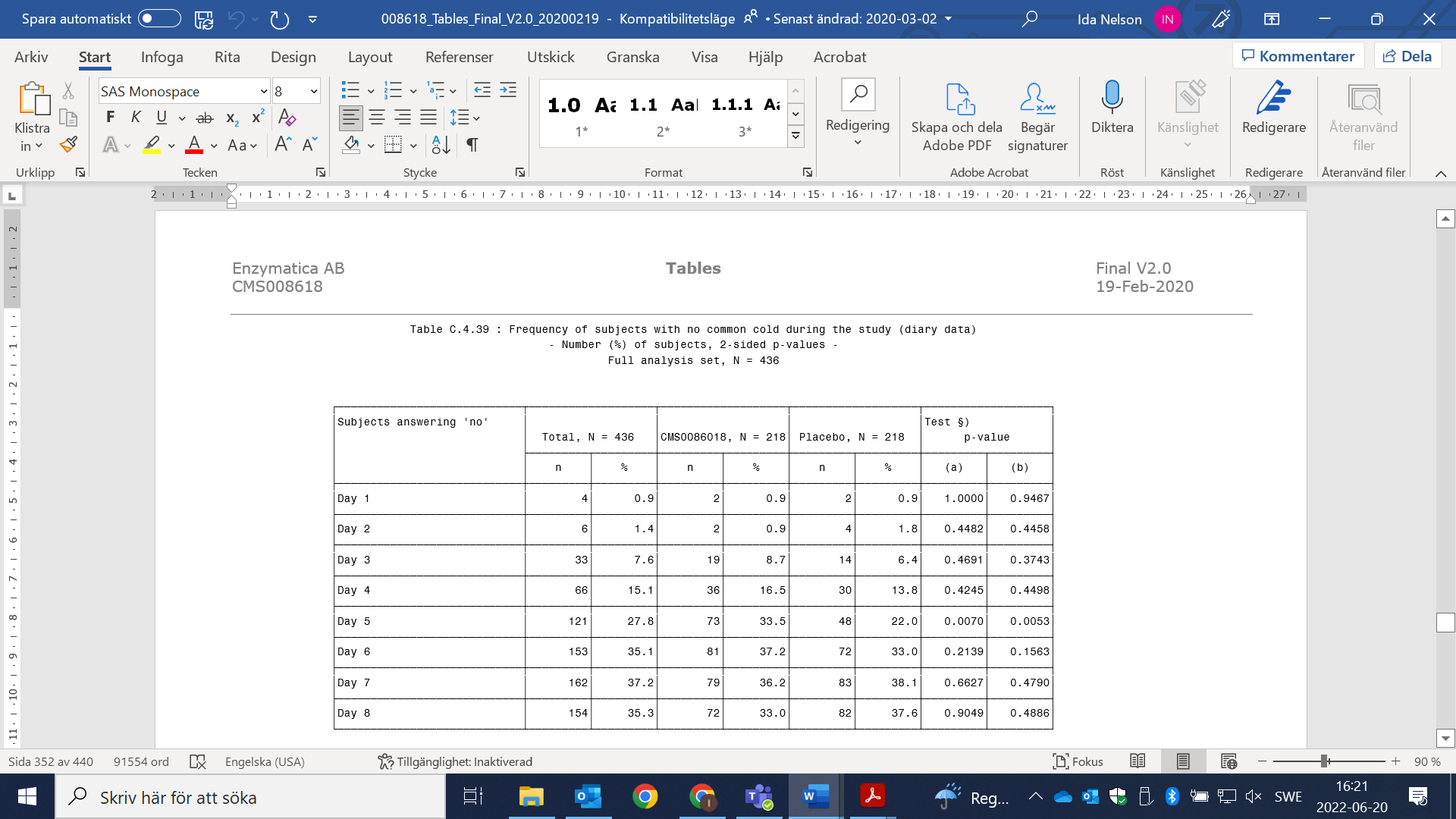


- Data reported in row 314. Reference: Table C.4.34

- Data reported in row 317. Reference: Table C.4.40


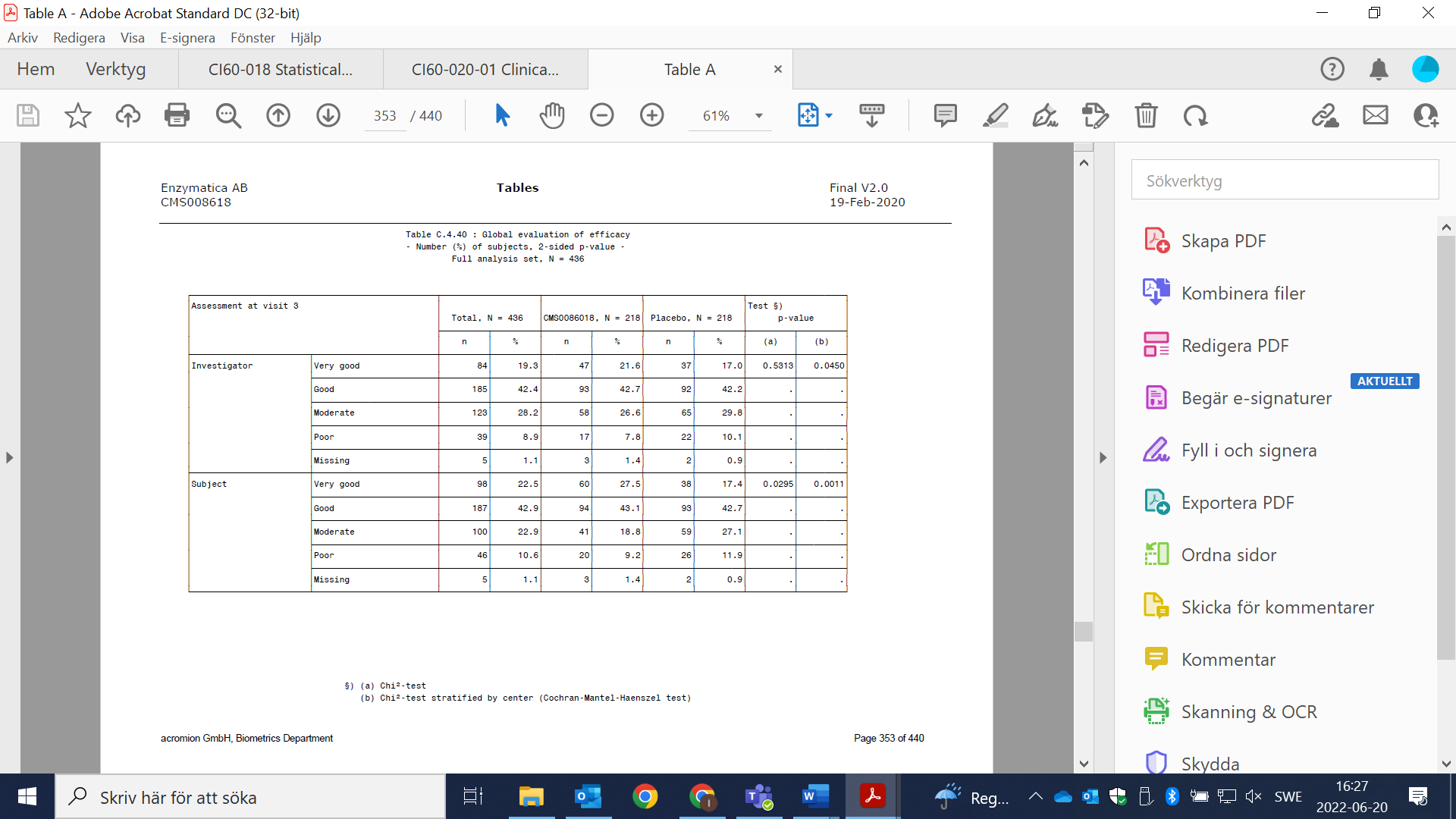


- Data reported in row 336. Reference: Table B.5.2.1.1.1


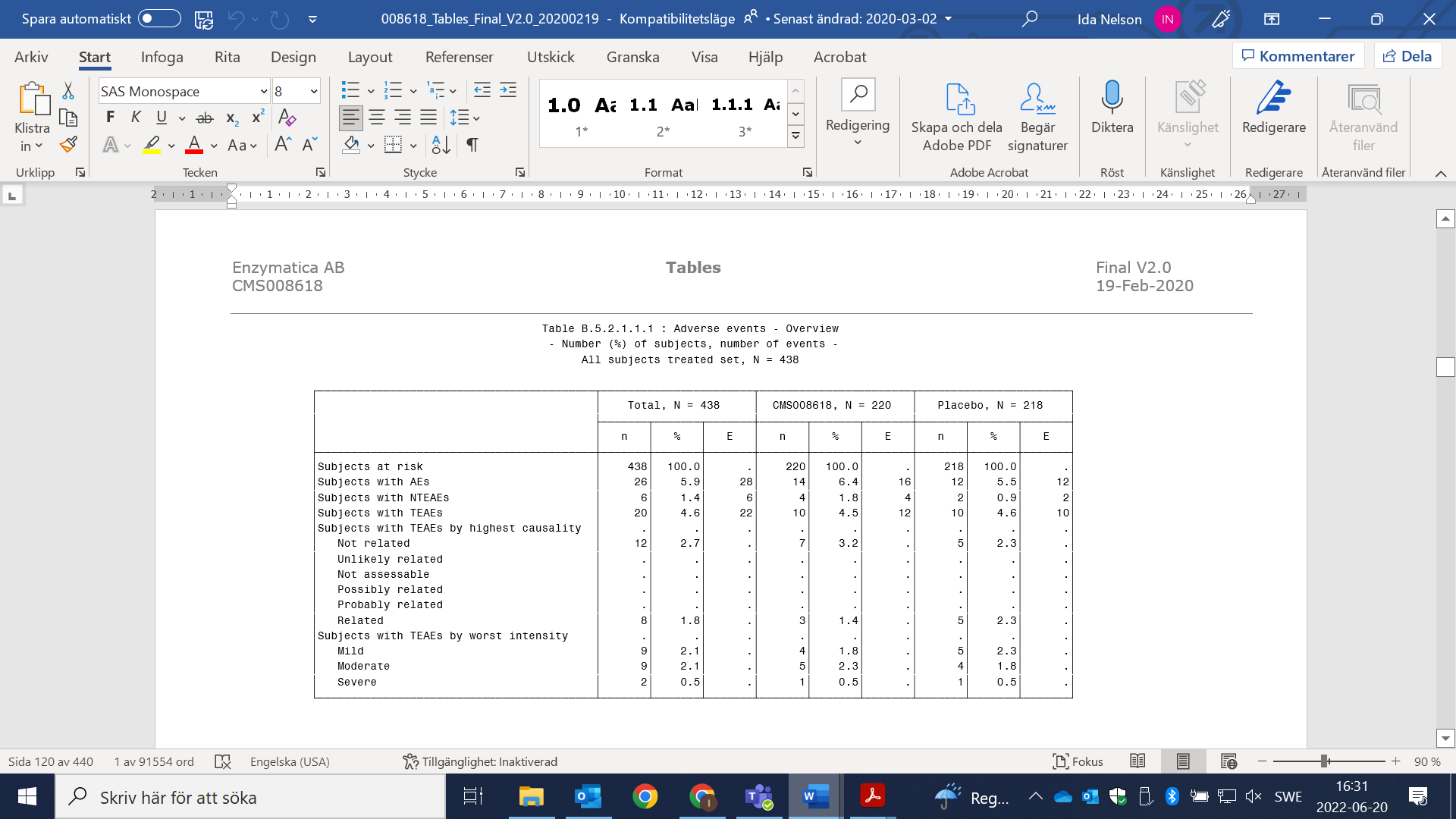


- Data reported in row 344. Reference: Table B.5.7
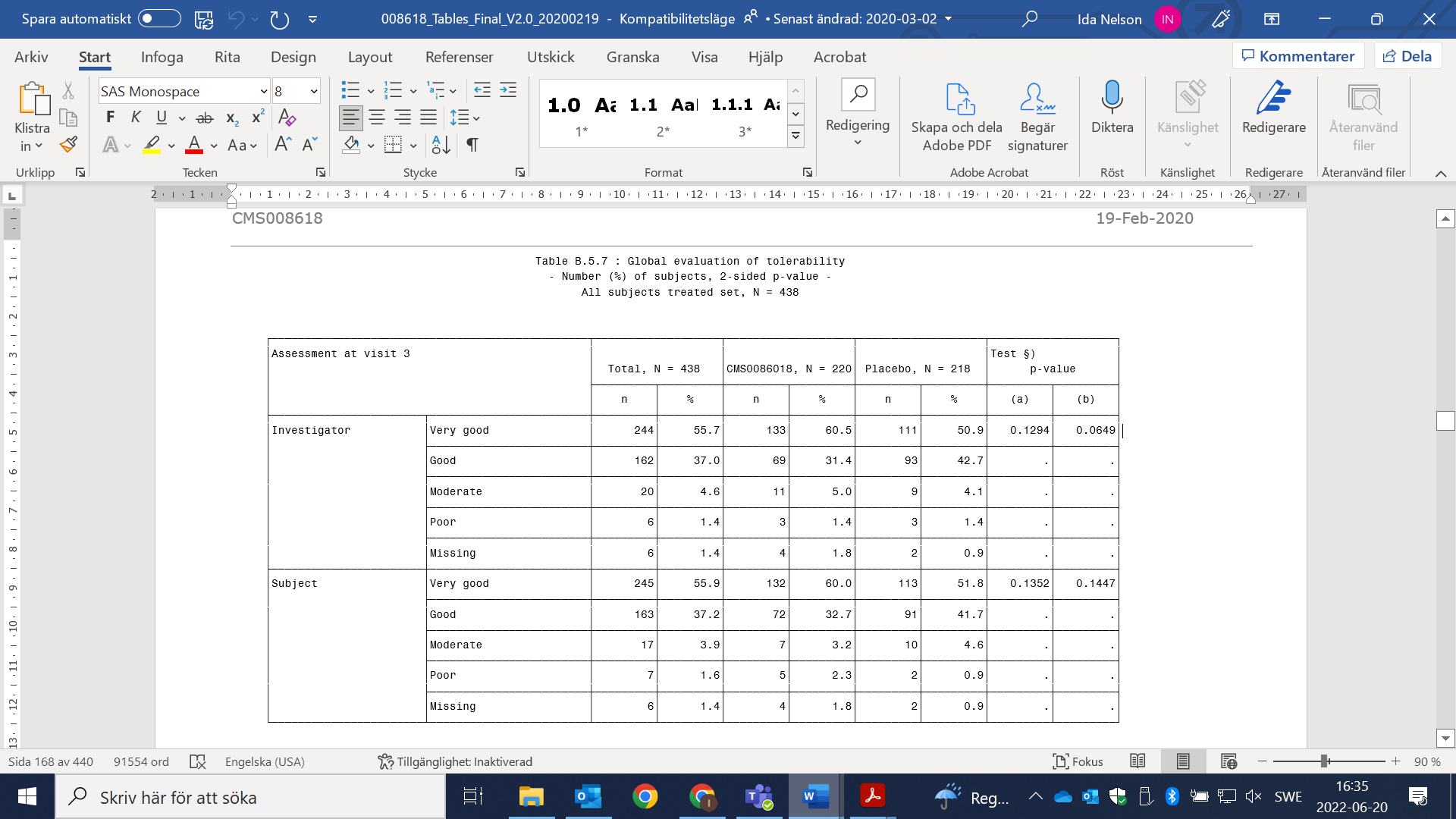


**Figure 1**

Based on the format of the CONSORT Flow Diagram, adapted from Table 19.1.1.1 in CI60-018.

| **Total,**  **N = 701** | | | **CMS008618,**  **N = 351** | | **Placebo,**  **N = 350** | |
| --- | --- | --- | --- | --- | --- | --- |
|  | n | % | n | % | n | % |
| **Subjects randomized** | 701 | 100.0 | 351 | 100.0 | 350 | 100.0 |
| **Subjects with symptoms of common cold** | 438 | 62.5 | 220 | 62.7 | 218 | 62.3 |
| **Subjects treated, thereof:** | 438 | 62.5 | 220 | 62.7 | 218 | 62.3 |
| **- Premature study termination** | 6 | 0.9 | 4 | 1.1 | 2 | 0.6 |
| **- Completion of study according to protocol** | 432 | 61.6 | 216 | 61.5 | 216 | 61.7 |
| **Subjects without symptoms of common cold** | 263 | 37.5 | 131 | 37.3 | 132 | 37.7 |
| **Subjects not treated, thereof:** | 263 | 37.5 | 131 | 37.3 | 132 | 37.7 |
| **- Premature study termination** | 14 | 2.0 | 3 | 0.9 | 11 | 3.1 |
| **- Completion of study according to protocol** | 249 | 35.5 | 128 | 36.5 | 121 | 34.6 |

**Table 1**

Reference: Table 19.2.1.1 in CI60-018 and Appendix Table C.2.3.3

| **Total,**  **N = 436** | | | | **CMS008618,**  **N = 218** | | **Placebo,**  **N = 218** | |
| --- | --- | --- | --- | --- | --- | --- | --- |
|  |  | n | % | n | % | n | % |
| **Gender** | Male | 139 | 31.9 | 65 | 29.8 | 74 | 33.9 |
|  | Female | 297 | 68.1 | 153 | 70.2 | 144 | 66.1 |
| **Age class** | 18 - 40 years | 223 | 51.1 | 114 | 52.3 | 109 | 50.0 |
|  | 41 - 55 years | 121 | 27.8 | 64 | 29.4 | 57 | 26.1 |
|  | 56 - 70 years | 92 | 21.1 | 40 | 18.3 | 52 | 23.9 |
| **Age (years)** | n | 436 |  | 218 |  | 218 |  |
|  | Mean | 41.3 |  | 40.9 |  | 41.7 |  |
|  | SD | 14.4 |  | 14.1 |  | 14.6 |  |
|  | Minimum | 18 |  | 18 |  | 18 |  |
|  | Median | 40.0 |  | 39.0 |  | 40.5 |  |
|  | Maximum | 70 |  | 68 |  | 70 |  |
| **Ethnicity** | Caucasian | 429 | 98.4 | 215 | 98.6 | 214 | 98.2 |
|  | Asian | 2 | 0.5 | 1 | 0.5 | 1 | 0.5 |
|  | Other | 5 | 1.1 | 2 | 0.9 | 3 | 1.4 |


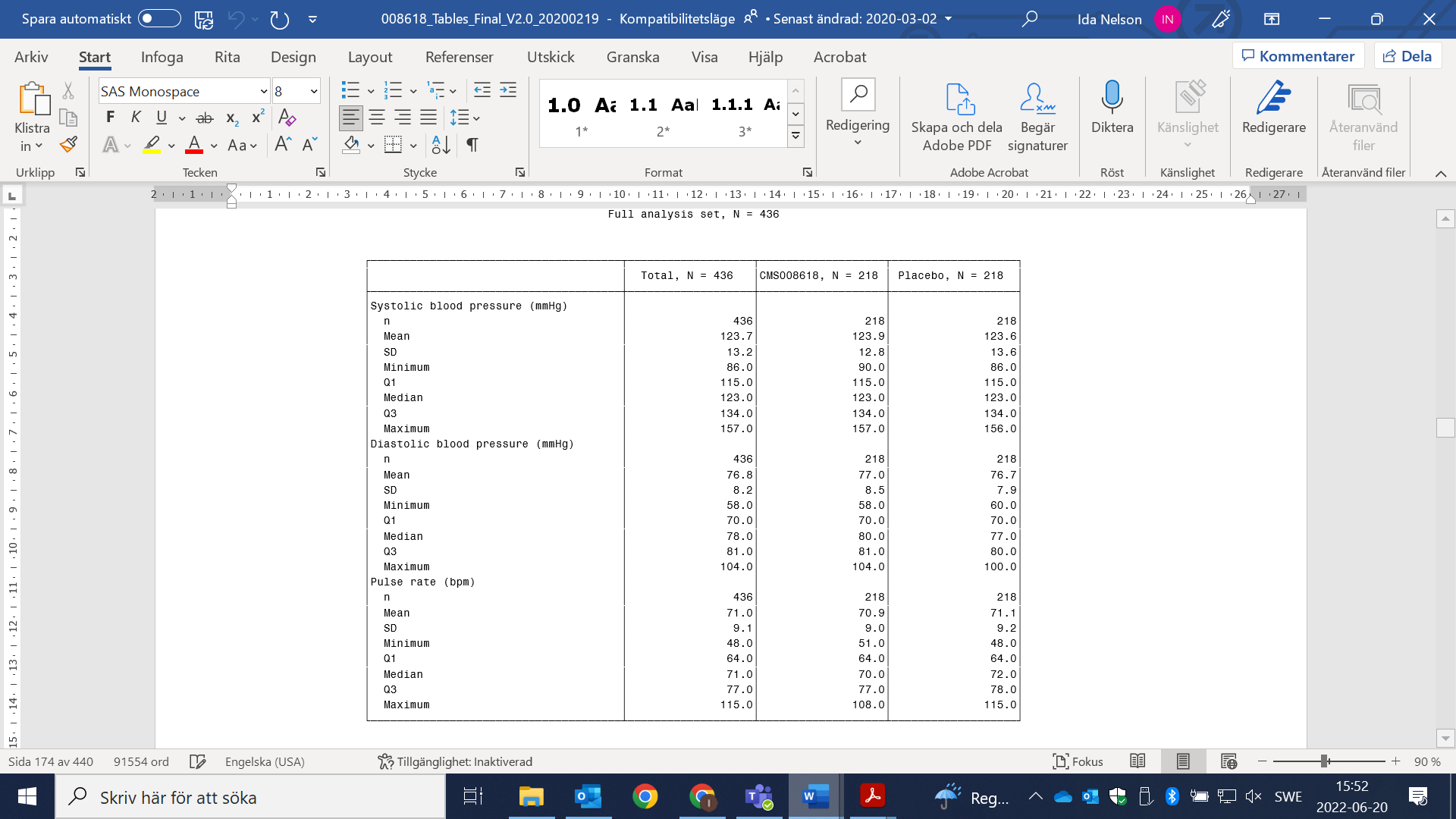

Supplement: S1 File — (DOCX) [file pone.0279204.s002.docx]
