# Supplementary material for: Early intervention with ColdZyme mouth spray after self-diagnosis of common cold: A randomized, double-blind, placebo-controlled study
Source: PLoS One. 2023 Jan 18;18(1):e0279204. doi: 10.1371/journal.pone.0279204 (PMC9847898; doi:10.1371/journal.pone.0279204)
Supplement: S2 File — (PDF) [file pone.0279204.s003.pdf]

**Clinical investigation plan**	
Investigational Study Product	CMS008618
Study Code	008618
Version	1.2
Date	28-MAY-2019

Double-blind, randomized, parallel-group, placebo-controlled study to evaluate efficacy of CMS008618 for common cold

The information in this clinical investigation plan is to be treated confidentially. They are only for information of the investigator, the study personnel and the Ethics Committee.

Document versions

Version	Date	Author	Comments
1.0	25-OCT-2018	Gordana Bothe, PhD	
1.1	04-DEC-2018	Gordana Bothe, PhD	Changes in biometry due to EC requirements
1.2	28-MAY-2019	Gordana Bothe, PhD	Refinements of endpoints

1 TABLE OF CONTENTS

1 TABLE OF CONTENTS 3
2 SYNOPSIS 7
3 LIST OF TERMS AND ABBREVIATIONS 14
4 INTRODUCTION 15
5 IDENTIFICATION AND DESCRIPTION OF THE INVESTIGATIONAL STUDY PRODUCT 16
5.1 DESCRIPTION OF THE INVESTIGATIONAL STUDY PRODUCT 16
5.2 MANUFACTURER OF THE IP 16
5.3 TRACEABILITY 16
5.4 INTENDED PURPOSE OF THE IP IN THE PROPOSED CLINICAL INVESTIGATION 16
5.5 POPULATIONS AND INDICATIONS FOR WHICH THE IP IS INTENDED 16
5.6 INSTRUCTION FOR USE 17
6 LITERATURE REVIEW 18
6.1 PRECLINICAL TESTING 18
6.2 CLINICAL DATA 18
6.3 JUSTIFICATION FOR THE DESIGN OF THE CLINICAL INVESTIGATION 19
7 RISK ASSESSMENT FOR THE INVESTIGATIONAL STUDY PRODUCT AND THE CLINICAL INVESTIGATION 20
7.1 PROSPECTIVE CLINICAL BENEFIT 20
7.2 ANTICIPATED ADVERSE DEVICE EFFECTS 20
7.3 RISK ANALYSIS 20
7.3.1 Product Specific Risks 20
7.3.2 Risks due to Manufacturing Errors 21
7.3.3 Risks due to Overdose 21
7.3.4 Risks due to Improper Use 21
7.3.5 Risks due to Contraindications 21
7.3.6 Overall risk Assessment 21
7.4 RISKS ASSOCIATED WITH THE PARTICIPATION IN THE CLINICAL INVESTIGATION 21
7.5 RISK-TO-BENEFIT RATIO 21
8 OBJECTIVES OF THE CLINICAL INVESTIGATION 22
8.1 PRIMARY ENDPOINT 22
8.2 SECONDARY ENDPOINTS 22
8.3 EXPLORATORY AND EXPLANATORY ENDPOINTS 22
8.4 SAFETY ENDPOINTS 23
9 DESIGN AND DURATION OF THE CLINICAL INVESTIGATION 24
9.1 DESIGN OF THE CLINICAL INVESTIGATION 24
9.2 DURATION OF THE STUDY 24
10 SELECTION OF STUDY SUBJECTS 25
10.1 NUMBER OF SUBJECTS 25
10.2 INCLUSION CRITERIA 25
10.3 CRITERIA FOR CONTINUATION AT V2 25
10.4 EXCLUSION CRITERIA 25
10.5 WOMEN OF CHILD BEARING POTENTIAL 26

11 CLINICAL INVESTIGATION TREATMENT 27
11.1 INVESTIGATIONAL STUDY PRODUCT 27
11.2 PLACEBO 27
11.3 DOSAGE AND USE OF IP 27
11.4 PRODUCTION, PACKAGING AND LABELLING OF IP 27
11.5 STORAGE, INVENTORY, RETURN, TRACEABILITY AND DOCUMENTATION OF THE INVESTIGATIONAL PRODUCT 28
11.6 METHODS OF RANDOMISATION AND BLINDING 29
11.7 BLINDING AND EMERGENCY ENVELOPES 29
11.8 UNBLINDING AND PROCEDURES FOR BREAKING CODES 29
11.9 COMPLIANCE 29
11.9.1 Overall Compliance to IP use 29
11.9.2 Compliance at the Start of IP use 30
11.9.3 Compliance at the Start of Filling out the Subject Cold Diary 30

12 CONCOMITANT TREATMENT 31

13 CONDUCT OF THE CLINICAL INVESTIGATION 32
13.1 VISIT 1 (V1, SCREENING, RANDOMISATION) – ALL SUBJECTS 32
13.2 VISITS FOR SUBJECTS WITH SYMPTOMS 32
13.2.1 Visit 2 (V2), within 1– 3 Days After Symptom Start 32
13.2.2 Visit 3 (V3, Final), 16 ± 4 Days After Symptom Start 32
13.3 VISIT FOR SUBJECTS WITHOUT SYMPTOMS DURING THE STUDY PERIOD: TERMINATION VISIT (TV), 16 WEEKS ± 7 DAYS AFTER V1 33
13.4 DEMOGRAPHIC AND ANTHROPOMETRIC DATA 33
13.5 MEDICAL HISTORY / PHYSICAL EXAMINATION 33
13.6 ASSESSMENT OF EFFICACY 33
13.6.1 Subject Diary 33
13.6.2 Common cold assessment at V2 35
13.6.3 Global Evaluation of Efficacy by the Subjects and Investigators 36
13.7 ASSESSMENT OF SAFETY 36
13.7.1 Blood Pressure and Pulse Rate 36
13.7.2 Adverse Events 36
13.7.3 Global Evaluation of Tolerability by the Subjects and Investigators 36
13.8 STUDY DISCONTINUATION 36
13.8.1 Withdrawal of Subjects 36
13.8.2 Study Discontinuation Criteria 37

14 SAFETY EVALUATION AND REPORTING 38
14.1 DEFINITIONS 38
14.1.1 Adverse Event (AE) 38
14.1.2 Adverse Device Effect (ADE) 38
14.1.3 Device Deficiency 38
14.1.4 Serious Adverse Event (SAE) 38
14.1.5 Serious Adverse Device Effect (SADE) 38
14.1.6 Unanticipated Serious Adverse Device Effect (USADE) 38
14.1.7 Incidents 39
14.2 DOCUMENTING AND REPORTING ADVERSE EVENTS, ADVERSE DEVICE EFFECTS AND DEVICE DEFICIENCIES 39
14.2.1 Documenting Adverse Events and Adverse Device Effects by the Investigator 39

14.2.2 Documenting and Reporting Device Deficiencies and Incidents by the Investigator 39
14.2.3 Reporting Serious Adverse Events by the Investigator 40
14.2.4 Safety Evaluation and Reporting by the Sponsor 40

15 STATISTICS 41
15.1 STATISTICAL HYPOTHESIS 41
15.2 SIGNIFICANCE LEVEL 41
15.3 SAMPLE SIZE CALCULATION 41
15.4 DROP-OUT RATE 42
15.5 ANALYSIS SETS 42
15.6 ELIGIBILITY, PROTOCOL DEVIATIONS 43
15.7 DEMOGRAPHICS AND OTHER BASELINE CHARACTERISTICS 43
15.8 ASSESSMENT OF COMPLIANCE 44
15.9 ANALYSIS OF PRIMARY ENDPOINT 44
15.10 ANALYSIS OF SECONDARY ENDPOINTS 44
15.11 ANALYSIS OF NON-CONFIRMATORY ENDPOINTS 45
15.12 ANALYSIS OF SAFETY ENDPOINTS 45
15.13 INTERIM ANALYSIS 46
15.14 SUBGROUP AND SENSITIVITY ANALYSIS 46
15.15 MISSING DATA 46
15.16 STATISTICAL ANALYSIS PLAN 46

16 ETHICAL AND LEGAL CONSIDERATIONS 47
16.1 REGULATORY ASPECTS 47
16.2 INDEPENDENT ETHICS COMMITTEE 47
16.3 SUBJECT INFORMATION AND INFORMED CONSENT FORM 47
16.4 INVESTIGATOR'S OBLIGATIONS 47
16.5 AMENDMENTS TO THE CIP 48
16.6 DATA PROTECTION AND CONFIDENTIALITY 48

17 QUALITY CONTROL AND QUALITY ASSURANCE 49
17.1 TRAINING 49
17.2 MONITORING 49
17.3 CIP DEVIATIONS 49
17.4 AUDITS AND INSPECTIONS 49

18 DATA HANDLING AND RECORD KEEPING 50
18.1 SOURCE DATA 50
18.2 DATA DOCUMENTATION 50
18.3 INVESTIGATOR SITE FILE 50
18.4 DATA MANAGEMENT 51
18.5 ARCHIVING 51
18.6 PRESENTATION OF DATA – FINAL REPORT 51

19 FINANCING / INSURANCE 52

20 PUBLICATION POLICY 53

21 LITERATURE 54

22 SIGNATURES 56
22.1 STUDY ADMINISTRATIVE STRUCTURE 57
22.2 STUDY FLOW CHART 58

22.2.1 Subjects with symptoms 58
22.2.2 Subjects without symptoms 59

2 SYNOPSIS

Title of the clinical investigation:	Double-blind, randomized, parallel-group, placebo-controlled study to evaluate efficacy of CMS008618 for common cold
Study code:	008618
Type of clinical investigation:	Double-blind, randomized, parallel-group design study according to German Act on Medical Devices (MPG) §23b
Investigational products (IP):	CMS008618 is ColdZyme® Mouth Spray, a marketed Class I medical device, with the following composition of the spray solution: glycerol, water, cod trypsin, ethanol (<1 %), calcium chloride, trometamol and menthol.<br>Placebo has the following composition of the spray solution: ethanol (<1 %), menthol and water.
Medical expert/ Coordinating investigator:	Prof. Ralf Uebelhack, MD<br>analyze & realize GmbH<br>Waldseeweg 6, 13467 Berlin, Germany<br>Phone: +49 30/40008 105<br>Fax: +49 30/40008 501<br>Email: ruebelhack@analyze-realize.com
Sponsor:	Ida Nelson<br>Enzymatica AB<br>Ideon Science Park, 22370 Lund, Sweden<br>Phone: +46 46 286 31 00<br>Email: ida.nelson@enzymatica.com
CRO:	analyze & realize GmbH<br>Waldseeweg 6<br>13467 Berlin, Germany<br><br>Project manager<br>Stephanie Seibt
Investigation site	analyze & realize GmbH<br>Weißenseerweg 111, 10369 Berlin, Germany
Clinical investigation objective:	The aim of the study is to evaluate the efficacy of CMS008618 for common cold, with the study hypothesis that treatment with CMS008618 is superior to placebo in the treatment of naturally occurring common cold. Thereby, the primary objective is to evaluate the impact on quality of life during a common cold episode, based on the primary endpoint defined as the area under the curve (AUC) of Wisconsin Upper Respiratory Symptom Survey (WURSS-21) Quality of Life (QoL) composite subscore during 8 days following start of symptoms (day 1 is the first day of symptom recording).
Proposed start of the study (first subject in):	Q4 2018
Proposed end of the study	Q3 2019

(last subject out):	
Visit schedule:	At screening visit (visit 1, V1), eligible subjects will be randomised to the study groups (CMS008618 and placebo). All subjects will get the subject diary.<br><br>Within 1-3 days after answering “yes” to the question on common cold and the onset of at least mild symptom(s) (except headache) in Jackson scale in the subject diary (see “Duration of intervention”) as well as the first IP use, the subject should attend visit 2 on site (V2). Between V1 and V2, the study centre will have monthly phone contact with the subjects as a reminder about the study. The final visit (V3) will take place on day 16 (± 4 days) after symptom start.<br><br>Subject who completed V1 but had no symptoms during the entire study period will only have the termination visit (TV) at 16 weeks (± 7 days) after V1.
Duration of intervention:	The IP use should start when the following conditions have been fulfilled:<br>• answering "Yes" to either of the questions in the subject diary: "Do you think/feel you have a cold?" or "Do you think/feel you are coming down with a cold (might be having first signs of cold)?"<br>• Jackson score of at least 1 (mild = present, but not disturbing or irritating) for any symptom except headache<br><br>The IP should be used until 2 days after the subject is symptom free (=answering "No" to the question "Do you think that you are still sick with this respiratory infection?" in the subject diary, for 2 days in a row), but not longer than 10 days in total.<br><br>The “rescue” treatment during the common cold period/start of IP use until V3 should be confined to:<br>• paracetamol (max. 2 g/day),<br>• ibuprofen (max. 400 mg/day),<br>• saline nose drops or nose spray<br>• as well as antibiotics (in case they are medically required for another ailment following the confirmation of the bacterial infection, however not to be used for common cold).
Duration of study:	Duration of study for each subject depends on the time point of symptom onset (see “Visit schedule”) and may be maximally 16 weeks (± 7 days) (in case of no symptoms).
Number of participants:	Expected number of subjects to be allocated to trial/randomised: n=600<br>Required number of subjects to enter the treatment phase (upon experiencing symptoms) and complete V2 and V3: n=400<br>Depending on the percentage of subjects from those randomized at V1 who actually develop symptoms during the study pe-

	riod, the envisaged number of 600 randomised subjects may deviate from the final number of randomised subjects needed.<br>For reasons of practicability/logistics of recruitment, the targeted number of subjects with symptoms (attending V2 and V3) may be subject to deviations.
Inclusion criteria:	1. Men and women<br>2. Age 18 to 70 years old<br>3. Increased risk for common cold (at least 3 self-reported occurences of common cold within the last 12 months prior to V1) but generally in good health<br>4. Readiness to comply with trial procedures, including in particular:<br>• Use of IP as recommended<br>• Filling in diary<br>• Keeping habitual life-style, including diet and physical activity level<br>• No use of products that may influence the study outcome (e.g. immune suppressants/immune stimulants including natural health products, analgesics/anti-rheumatics, anti-phlogistics, anti-tussives/expectorants, mouth or throat therapeutics, decongestants, antibiotics, anti-histaminergic drugs, nasal drops/spray) during the study (except for the defined “rescue” treatment)<br>5. Women of child-bearing potential:<br>• Have to agree to use appropriate contraception methods<br>• Negative pregnancy testing (beta human chorionic gonadotropin test in urine) at V1<br>Participation is based upon written informed consent by the participant following written and oral information by the investigator regarding nature, purpose, consequences and possible risks of the clinical study.
Criteria for continuation at V2:	Subjects having Visit 2 after onset of common cold symptoms and start of IP use (according to predefined criteria) will continue study participation.
Exclusion criteria:	1. Known allergy or hypersensitivity to the components of the investigational product<br>2. History and/or presence of clinically significant condition/ disorder (self-reported), which per investigator's judgement could interfere with the results of the study or the safety of the subject, e.g.:<br>• Nasal disorder (e.g. polyposis, relevant septal deviation, ulcer etc.) and/or reconstructive surgery<br>• Asthma, chronic obstructive lung disease or any other acute/chronic airways disease/disorder (e.g. chronic cough of any origin)<br>• Acute psychiatric disorders<br>• Any other acute/chronic serious organ or systemic diseases<br>3. Influenza vaccination within the last 3 months prior to V1

	and during the study<br>4. Regular use of products that may influence the study outcome (e.g. immune suppressants/immune stimulants including natural health products, analgesics/anti-rheumatics, antiphlogistics, antitussives/expectorants, mouth or throat therapeutics, decongestants, antibiotics, anti-histaminergic drugs, nasal drops/spray) within the last 4 weeks prior to V1<br>5. Pregnancy or nursing<br>6. History of (in the past 12 months prior to V1) or current abuse of drugs, alcohol or medication<br>7. Participation in the present study of a person living in the same household as the subject<br>8. Inability to comply with study requirements according to investigator's judgement<br>9. Participation in another clinical study in the 30 days prior to V1 and during the study
Investigational product use:	The IP should be applied every second hour up to 6 times daily, each time 2 sprays (1 dose).<br><br>Depending on the timepoint of treatment onset, there may be less IP applied on the first day of use.
Assessments:	• Physical examination, vital signs (blood pressure/ pulse measurement) at V1, V2 and V3<br>• Subject diary comprising:<br>- Daily diary, questioning on possible presence/absence of a common cold (twice daily, morning/evening) from the study start on<br>- Cold diary, questioning on the following items from the onset of symptoms on:<br>o Sore Throat Scale (once daily, in the evening)<br>o Jackson scale (twice daily, morning/evening)<br>o Wisconsin Upper Respiratory Symptom Survey (WURSS-21) Quality of Life (QoL) (once daily, in the evening)<br>o Use of the IP<br>o Use of any concomitant treatment / remedies<br>• Examination by investigator at V2 incl. assessment of symptoms (common cold/another disorder)<br>• Assessment of adverse events at all visits<br>• Assessment of device deficiencies at V2 and V3<br>• Urinalysis at V1<br>• Global evaluation of efficacy by subjects and investigators (4-point categorical scale) at V3<br>• Global evaluation of tolerability by subjects and investigators (4- point categorical scale) at V3
Primary endpoint (in	WURSS-21 QoL composite subscore AUC days 1-8

comparison between verum and placebo):	(day 1 is the first day of symptom recording)
Major secondary endpoints (in comparison between verum and placebo):	• AUC days 1-8 composite daily severity of all symptoms within the Jackson score (mean of morning and evening) (day 1 is the first day of symptom recording)<br>• Exposure to any concomitant treatment (including natural health products) that may affect common cold symptoms - immune suppressants/immune stimulants, analgesics/anti-rheumatics, anti-phlogistics, antitussives/expectorants, mouth or throat therapeutics, decongestants, antibiotics, anti-histaminergic drugs, nasal drops/spray or any medication/treatment known to affect common cold symptoms - at any dose, expressed as number of days with concomitant treatment during the first 4 days for each subject (based on diary data).
Other secondary endpoints (in comparison between verum and placebo):	• AUC days 1-8 for each single WURSS-21 QoL subscore item<br>• AUC days 1-8 composite daily severity of all local symptoms within the Jackson score (mean of morning and evening)<br>• AUC days 1-8 composite daily severity of each individual symptom of the Jackson score (mean of morning and evening)<br>• Frequency of subjects with use of concomitant treatment (including natural health products) that may affect common cold symptoms - immune suppressants/immune stimulants, analgesics/anti-rheumatics, anti-phlogistics, antitussives/expectorants, mouth or throat therapeutics, decongestants, antibiotics, anti-histaminergic drugs, nasal drops/spray or any medication/treatment known to affect common cold symptoms - at any dose, during days 1-4 (based on diary data)<br>• Assessment of duration of first intense phase, expressed as number of days from start of treatment until scoring <5 in total Jackson score<br>• Assessment of symptom intensity, expressed as mean total Jackson score days 1-4<br>• Assessment of symptom sore throat per Sore Throat Scale, expressed as AUC days 1-8<br>• Percentage of subjects with confirmed common cold at Visit 2 (from all subjects with V2)<br>• Global evaluation of efficacy by subjects and investigators at study end

Safety endpoints:	• Physical examination throughout the study<br>• Vital signs (blood pressure, pulse rate) throughout the study<br>• Global evaluation of tolerability by subjects and investigators at study end<br>• Assessment of adverse events throughout the study<br>• Assessment of device deficiencies at V2 and V3
Biometry:	The statistical analyses will be confirmatory for the primary endpoint and the major secondary endpoints, and exploratory for all other secondary endpoints. All statistical testing will performed two-sided. The confirmatory significance level is fixed to a type I error rate alpha of 5% (two-sided). In order to control the overall type I error rate, the primary endpoint and the two major secondary endpoints will be tested in a fixed sequence as a-priori ordered hypotheses. That is, the first major secondary endpoint will be tested only if the primary endpoint is positive (i.e. $p <0.05$), and the second major secondary endpoint will be tested only if the first major secondary endpoint is positive.<br><br>The primary endpoint is defined as the AUC of WURSS-21 QoL composite subscores assessed during first 8 days of symptoms. The WURSS-21 QoL composite subscore is calculated by summing the scores of the 9 consecutive items of WURSS-21, from item 12 (“think clearly”) to item 20 (“live your personal life”), as documented in the subject diary (see 13.6.1). The AUC will be assessed by applying the trapezoidal approximation. The two-sided confirmatory null-hypothesis for the primary endpoint will be tested with the non-parametric two-sided Wilcoxon (Mann-Whitney) rank-sum test.<br><br>The major secondary endpoints are defined as (1 is highest ranking):<br>1. AUC days 1-8 composite daily severity of all symptoms within the Jackson score (mean of morning and evening) (day 1 is the first day of symptom recording)<br>2. Exposure to any concomitant treatment (including natural health products) that may affect common cold symptoms - immune suppressants/immune stimulants, analgesics/anti-rheumatics, anti-phlogistics, antitussives/expectorants, mouth or throat therapeutics, decongestants, antibiotics, anti-histaminergic drugs, nasal drops/spray or any medication/treatment known to affect common cold symptoms - at any dose, expressed as number of days with concomitant treatment during the first 4 days for each subject (based on diary data).<br><br>The primary endpoint and the major secondary endpoints are ranked according to clinical relevance. No confirmatory claims will be based on endpoints that have a rank lower than or equal to that variable whose null hypothesis is the first that could not

	be rejected (Points to consider on multiplicity issues in clinical trials, CPMP/EWP/908/99, 2002).<br>The composite Jackson score is calculated by summing the scores of 8 items, as documented in the subject diary (see 13.6.1). The AUC will be assessed by applying the trapezoidal approximation. The two-sided confirmatory null-hypothesis for the first major secondary endpoint will be tested with the non-parametric two-sided Wilcoxon (Mann-Whitney) rank-sum test.<br>The use of concomitant treatment is calculated as the number of days with concomitant treatment during the first 4 days for each subject (based on diary data), as documented in the subject diary (see 13.6.1). The two-sided confirmatory null-hypothesis for the second major secondary endpoint will be tested using the non-parametric two-sided Wilcoxon (Mann-Whitney) rank-sum test.<br>Data of the major secondary endpoints will be summarized by descriptive statistics using appropriate tabulation.<br>All other secondary endpoints will be analyzed exploratory only and will be descriptively assessed. For continuous data, standard statistical characteristics will be presented (number of subjects with non-missing data, mean, standard deviation, median, minimum and maximum and quartiles). Categorical data (nominal or ordinal) will be summarized using frequency tables. Continuous endpoints will be analyzed for between-group comparison using the non-parametric two-sided Mann-Whitney-U-test. Multiple testing will be performed without exploratory adjustment for multiple testing. For categorical endpoints chi² tests will be used for between-group comparison.<br>A more detailed description of the planned descriptive and exploratory analyses for all endpoints will be presented in the SAP.
GCP conformity:	This clinical investigation will be performed based on the principles of the ICH GCP E6 (R2) and ISO 14155.

3 LIST OF TERMS AND ABBREVIATIONS

AE	Adverse Event
ADE	Adverse Device Effect
BMI	Body Mass Index
CE	European Conformity (“Conformité Européenne”)
CHMP	Committee for Medicinal Products for Human Use
CIP	Clinical Investigation Plan
CRA	Clinical Research Associate
CRF	Case Report Form
CRO	Contract Research Organization
EC	Ethics Committee
FAS	Full Analysis Set
GCP	Good Clinical Practice
GI	Gastrointestinal
ICH	International Conference on Harmonization
ICF	Informed Consent Form
IP	Investigational Product
ISF	Investigator Site File
ITT	Intention To Treat
LSO	Last Subject Out
MPG	German Act on Medical Devices (“Medizinproduktegesetz”)
MPSV	Ordinance on Medical Devices Vigilance (“Medizinprodukte-Sicherheitsplanverordnung”)
PP	Per Protocol
SAE	Serious Adverse Event
SADE	Serious Adverse Device Effect
SAP	Statistical Analysis Plan
SOP	Standard Operating Procedure
TMF	Trial Master File
USADE	Unexpected Serious Adverse Device Effect
V	Visit
VCAS	Valid Case Analysis Set
WHO	World Health Organization
WURSS QoL	Wisconsin Upper Respiratory Symptom Survey Quality of Life

All official and professional titles and any references to persons in this document apply to both genders.

4 INTRODUCTION

A common cold is a self-limited viral infection of the upper respiratory tract (Tyrrell, 1996). Acute respiratory diseases are among the most frequent illnesses of all. They are almost exclusively caused by viruses and mostly show a natural history affecting all age groups, with well-known autumn/winter peaks (Wagner, 1996).

The typical progression differs in individuals, yet in all cases common cold presents a burden for the affected person's quality of life. Most commonly, adult subjects complain of a general feeling of sickness, nasal congestion, headache, rhinorrhea (runny nose), sore throat, hoarseness, cough and altered quality of sleep. In general, the symptoms reach maximal severity 2 to 3 days following infection, with an average overall duration of 7 to 10 days (Heikkinen & Jarvinen, 2003). A sore scratchy throat is most often a harbinger of the common cold appearing on the first day of illness; rhinorrhea and nasal congestion soon follow (Witek et al., 2015). A treatment used at the first sign of a cold that targets the scratchy upper pharynx and thereafter reduces throat and nasal symptoms, would be much desired.

Effective curative options are few due to the multiple virus types and the complex interaction between virus and host (Johnston, 1997; Hendley, 2008). Numerous symptomatic remedies are available over the counter, yet there is still no reliable treatment and their potential side effects may be substantial (Eccles et al., 2010). Effective solutions with minimal side effects (given the nonhazardous nature of the disorder) are required.

CMS008618 is ColdZyme® Mouth Spray, a marketed Class I medical device (Enzymatica AB, Sweden). It is to be used when exposed to cold viruses or early on when cold symptoms occur. It works by coating the lining of the mucous membrane, creating a protective barrier that acts osmotically on the cold viruses, trapping them and preventing them from binding with human cells, helping the body to remove them naturally.

Previous clinical experience with ColdZyme® Mouth Spray has shown promising results with respect to its cold-reducing effects (section 6 and Investigator's Brochure (IB), 2018). The present study aims at generating further supporting data on the efficacy of CMS008618 for common cold using the "gold-standard" design - a double-blind, placebo-controlled, randomized clinical investigation, in generally healthy subjects acquiring the common cold in their natural setting of everyday life.

5 IDENTIFICATION AND DESCRIPTION OF THE INVESTIGATIONAL STUDY PRODUCT

5.1 DESCRIPTION OF THE INVESTIGATIONAL STUDY PRODUCT

CMS008618 is ColdZyme® Mouth Spray, a medical device classified as a Class I device (CE-marked and marketed since 2013) under Council Directive 93/42/EEC on Medical Device and MED-DEV Guidance Document 2.4/1. Rev 8, June 2010, with the following composition of the spray solution: glycerol, purified water, cod trypsin, ethanol (<1 %), calcium chloride, trometamol and menthol.

ColdZyme® is a non-sterile medical device with the following components:

- A primary container consisting a 20 ml semi-transparent plastic bottle, pump, actuator (spray nozzle) and an actuator terminal cap; a thin plastic label is added to the bottle.
- A secondary container consisting of a paper box.

CMS008618 (ColdZyme®) is designed to deposit a viscous solution containing primarily glycerol and trypsin to the mouth/throat, resulting in a thin protective barrier formed on the oropharyngeal mucous membrane.

For further details, please refer to the IB (2018).

5.2 MANUFACTURER OF THE IP

Enzymatica AB
Ideon Science Park
223 70 Lund, Sweden

5.3 TRACEABILITY

To ensure traceability, there is a batch number system in place according to EN ISO 13485.

5.4 INTENDED PURPOSE OF THE IP IN THE PROPOSED CLINICAL INVESTIGATION

The intended use of ColdZyme® Mouth Spray (according to the IB, 2018):

- It can reduce the probability of catching a cold
- it can help shorten the duration of a cold if used at an early stage of the infection.

The present study focuses on generating clinical data with respect to cold symptoms when ColdZyme® (CMS008618) is used at an early stage of the infection.

5.5 POPULATIONS AND INDICATIONS FOR WHICH THE IP IS INTENDED

The indication for ColdZyme® Mouth Spray (according to the IB, 2018):

- To be used when exposed to cold viruses or early on when cold symptoms occur; it is suitable for adults and children over 4 years.

5.6 INSTRUCTION FOR USE

The IP should be applied every second hour up to 6 times, with each time 2 sprays (1 dose) per occasion. The user should open the mouth, aim the nozzle towards the throat, press on the pump and spray 2 times per occasion.

Figure 1: Application of spray

6 LITERATURE REVIEW

6.1 PRECLINICAL TESTING

In a recent non-clinical study (Stefansson et al, 2017), it was demonstrated that ColdZyme® Mouth Spray has a virus deactivating ability against four major common cold viruses. A virucidal efficacy suspension test was conducted using ColdZyme® Mouth Spray against each of the challenge viruses in suspension. ColdZyme® Mouth Spray deactivated rhinovirus type 1A by 91.7%, rhinovirus type 42 by 92.8%, human influenza A virus H3N2 by 96.9%, respiratory syncytial virus (RSV) by 99.9% and adenovirus type 2 by 64.5%.

A biological safety assessment has confirmed that no undue toxicological hazards are associated with the use of the materials of construction of ColdZyme® Mouth Spray and no appreciable toxicological risks are likely to arise from its intended use. The biological evaluation was conducted in accordance with relevant principles set out in ISO 10993-1:2010 and included chemical characterisation and appropriate biological tests (IB, 2018).

6.2 CLINICAL DATA

A recent clinical study (Clarsund et al., 2017) investigated the performance of ColdZyme® Mouth Spray in rhinovirus-inoculated healthy volunteers. This randomized, double-blind, placebo-controlled pilot study was conducted on 46 healthy volunteers inoculated with rhinovirus 16 via the nose. Subjects self-administered ColdZyme® or placebo 6 times daily for 11 days and recorded symptoms daily in a diary. Rhinovirus 16 in nasal and oropharyngeal samples at days 0, 3, 4, 6, 7 and 10 were assessed by quantitative real-time polymerase chain reaction. The primary outcome measure was the reduction in viral load in oropharyngeal samples. Exploratory analysis measuring the total viral load (i.e., area under the curve) for days 3-10 in successfully inoculated subjects found that ColdZyme® Mouth Spray treatment resulted in a lower total viral load in the oropharynx ($p = 0.023$). In subjects who experienced a symptomatic common cold, treatment with ColdZyme® Mouth Spray resulted in a reduction in the number of days with common cold symptoms from 6.5 to 3.0 days ($p = 0.014$) in comparison to placebo.

The safety and performance of ColdZyme® Mouth Spray was evaluated in further clinical investigations and post-marketing surveillance/user surveys, confirming that when ColdZyme® was used, colds contracted were of reduced duration and intensity (IB, 2018). The complication rate was comparable to that in a literature review of similar products.

Lastly, a recent exploratory methodological clinical study (sponsor's data on file) investigated the performance of ColdZyme® Mouth Spray in a population of 267 subjects with a common cold in real-life conditions. The subjects were randomised to two groups, the verum group receiving ColdZyme® and the "optional care only" group not receiving it. Both groups could use optional care as needed during the common cold period, comprising the following options: paracetamol (max. 2 g/day), ibuprofen

(max. 400 mg/day), sea water nose drops or nose spray as well as antibiotics (if required). The study showed significant effects of ColdZyme® Mouth Spray with respect to reduction of symptoms (both assessed by Jackson scale (Jackson et al., 1958) and the Sore/Irritated Throat Scales (Shephard et al., 2015).) and related functional impairment as rated per WURSS-21 Quality of Life subscore (Barrett et al., 2009).

With respect to use of the marketed product, a review of all complaints in the period from 2013 to January 2018 has been completed with an overall complaint rate of 0.014% and none of the complaints were judged to be reportable to authorities according to MEDDEV 2.12-1 rev8 (IB, 2018).

6.3 JUSTIFICATION FOR THE DESIGN OF THE CLINICAL INVESTIGATION

In accordance with the recommendations for assessment of efficacy as per the Note for Guidance on Good Clinical Practice CPMP/ICH/135/95, the study is designed as a randomized, placebo-controlled, double-blind clinical trial. A precedent randomized study (sponsor's data on file) evaluated Jackson symptom score scale, other symptom scales and WURSS-21 QoL functional parameters, and compared ColdZyme® with no treatment in naturally occurring common cold. All scales demonstrated an ability to detect a statistically significant benefit from the usage of ColdZyme® compared to no intervention, most sensitive being the WURSS-21 QoL scale. The result from this study justifies the use of WURSS-21 QoL scale as the primary endpoint in the current clinical investigation. The choice of placebo as a comparator is considered appropriate for the present study, as this is the first confirmatory clinical investigation with CMS008618 under real-life conditions.

As there is no adequate causal therapy for common cold currently available, the focus of clinical research in this indication lies in the evalution of symptom relief (Eccles, 2005). Thus, the principal assesments in the study have been selected accordingly. Thereby, subject-reported outcomes have been chosen as the main endpoints, since they are widely acknowledged as the most relevant options of symptom rating (Eccles, 2012). The study population and the specified timepoints of assessments (including symptom records in the very early phase of the cold) are aligned with the intended use labeling of ColdZyme® Mouth Spray (IB, 2018).

7 RISK ASSESSMENT FOR THE INVESTIGATIONAL STUDY PRODUCT AND THE CLINICAL INVESTIGATION

7.1 PROSPECTIVE CLINICAL BENEFIT

Based on the available clinical evidence, an improvement of common cold symptoms is anticipated within the period of treatment with CMS008618 (ColdZyme® Mouth Spray).

7.2 ANTICIPATED ADVERSE DEVICE EFFECTS

The essential requirements relevant for the device safety and performance have been assessed within an extensive risk management process, which incorporates biological evaluation, design, usability, production, quality and regulatory processes and clinical validation. Evaluation of the analysis combining all potential risks has concluded that the ColdZyme® Mouth Spray is free from unacceptable risk (IB, 2018). No contraindications for use of the device, other than hypersensitivity to any of the ingredients, have been identified in the risk management process. Risks have been further addressed in post-market surveillance and in post-market investigations. No new or unacceptable risks have been recorded, and no adverse events related to the use of the ColdZyme® Mouth Spray have been reported during post-market investigations. The chosen ingredients are well known and non-toxic. Trypsin, glycerol, calcium chloride, water, ethanol and menthol are listed in the “Generally Recognized As Safe“ (GRAS) substance database of the U. S. Food and Drug Administration.
Therefore, the device is concluded to achieve its intended purpose and fulfil essential requirements regarding safety and performance. For further details, please refer to the IB (2018).

The study subjects will be instructed on the relevant precautions applying to CMS008618 (ColdZyme® Mouth Spray), according to the IB (2018):

- It is not to be used by individuals hypersensitive/allergic to any of the ingredients
- The user should not inhale when applying the spray since this may cause transient asthma-like symptoms, such as coughing and hoarseness

7.3 RISK ANALYSIS

7.3.1 Product Specific Risks

Safety of ColdZyme® Mouth Spray has been assessed during the risk management process and evaluation of the analysis combining all potential risks has concluded that ColdZyme® Mouth Spray does not impose any unacceptable risk to the user. The essential requirements relevant for ColdZyme® Mouth Spray safety have been handled according to the Risk Management process. Risks have been identified and evaluated according to the essential requirements with applicable harmonized standards, mitigated and, where applicable, controlled via verified control measures.

7.3.2 Risks due to Manufacturing Errors

To mitigate the risk, the production is monitored by means of a quality management system ensuring that the quality of the manufactured product complies with the specified standards.

7.3.3 Risks due to Overdose

To mitigate the risk, the dosing recommendations for the intended use of the device are derived from the clinical evaluation of the product.

7.3.4 Risks due to Improper Use

There is labeling on the device packaging stating proper use and general and specific precautions including a 'use by date' statement and proper storage requirements. In the study, the subjects will be trained on proper use (please refer to section 11.3).

7.3.5 Risks due to Contraindications

ColdZyme® Mouth Spray should not be used by individuals hypersensitive/allergic to any of the ingredients.

7.3.6 Overall risk Assessment

According to the Risk Management Report, possible risks are acceptable due to low probability of occurrence and existence of sufficient countermeasures.

For further details, please refer to the IB (2018).

7.4 RISKS ASSOCIATED WITH THE PARTICIPATION IN THE CLINICAL INVESTIGATION

All clinical assessments are standard practice in health care as applied to upper respiratory tract infections.

7.5 RISK-TO-BENEFIT RATIO

The Risk Management Report confirms that all individual and cumulative risks associated with the use of this device have been adequately controlled and reduced to a level as low as possible (IB, 2018). It can be concluded that, since the current controls adequately ensure the safety of ColdZyme® Mouth Spray (CMS008618) and clinical studies have shown a beneficial effect on viral load and duration of symptoms, the risk-to-benefit-ratio in the present investigation may be considered as favourable.

8 OBJECTIVES OF THE CLINICAL INVESTIGATION

The aim of this double-blind, randomized, parallel-group design clinical investigation is to evaluate the efficacy of CMS008618 for common cold.

The endpoints selected to evaluate the effects of CMS008618 are based on the state-of-the-art assessments of upper respiratory tract infections.

8.1 PRIMARY ENDPOINT

Primary endpoint is the AUC of Wisconsin Upper Respiratory Symptom Survey (WURSS-21) Quality of Life composite subscore during first 8 days of symptoms, to be assessed in comparison between verum and placebo.

8.2 MAJOR SECONDARY ENDPOINTS

The following major secondary endpoints will be assessed in comparison between verum and placebo (1 is highest ranking):

1. AUC days 1-8 composite daily severity of all symptoms within the Jackson score (mean of morning and evening) (day 1 is the first day of symptom recording)
2. Exposure to any concomitant treatment (including natural health products) that may affect common cold symptoms - immune suppressants/immune stimulants, analgesics/anti-rheumatics, anti-phlogistics, antitussives/expectorants, mouth or throat therapeutics, decongestants, antibiotics, anti-histaminergic drugs, nasal drops/spray or any medication/treatment known to affect common cold symptoms - at any dose, expressed as number of days with concomitant treatment during the first 4 days for each subject (based on diary data).

8.3 OTHER SECONDARY ENDPOINTS

To be assessed in comparison between verum and placebo:

- AUC days 1-8 for each single WURSS-21 QoL subscore item
- AUC days 1-8 composite daily severity of all local symptoms within the Jackson score (mean of morning and evening)
- AUC days 1-8 composite daily severity of each individual symptom of the Jackson score (mean of morning and evening)
- Frequency of subjects with use of concomitant treatment (including natural health products) that may affect common cold symptoms - immune suppressants/immune stimulants, analgesics/anti-rheumatics, anti-phlogistics, antitussives/expectorants, mouth or throat therapeutics, decongestants, antibiotics, anti-histaminergic drugs, nasal drops/spray or any medication/treatment known to affect common cold symptoms - at any dose, during days 1-4 (based on diary data)

- Assessment of duration of first intense phase, expressed as number of days from start of treatment until scoring <5 in total Jackson score
- Assessment of symptom intensity, expressed as mean total Jackson score days 1-4
- Assessment of symptom sore throat per Sore Throat Scale, expressed as AUC days 1-8
- Percentage of subjects with confirmed common cold at Visit 2 (from all subjects with V2)
- Global evaluation of efficacy by subjects and investigators at study end

8.4 SAFETY ENDPOINTS

The following safety endpoints will be assessed:

- Physical examination throughout the study
- Vital signs (blood pressure, pulse rate) throughout the study
- Global evaluation of tolerability by subjects and investigators at study end
- Assessment of adverse events throughout the study
- Assessment of device deficiencies at V2 and V3

9 DESIGN AND DURATION OF THE CLINICAL INVESTIGATION

9.1 DESIGN OF THE CLINICAL INVESTIGATION

The present clinical investigation is a double-blind, randomized, parallel-group design study.

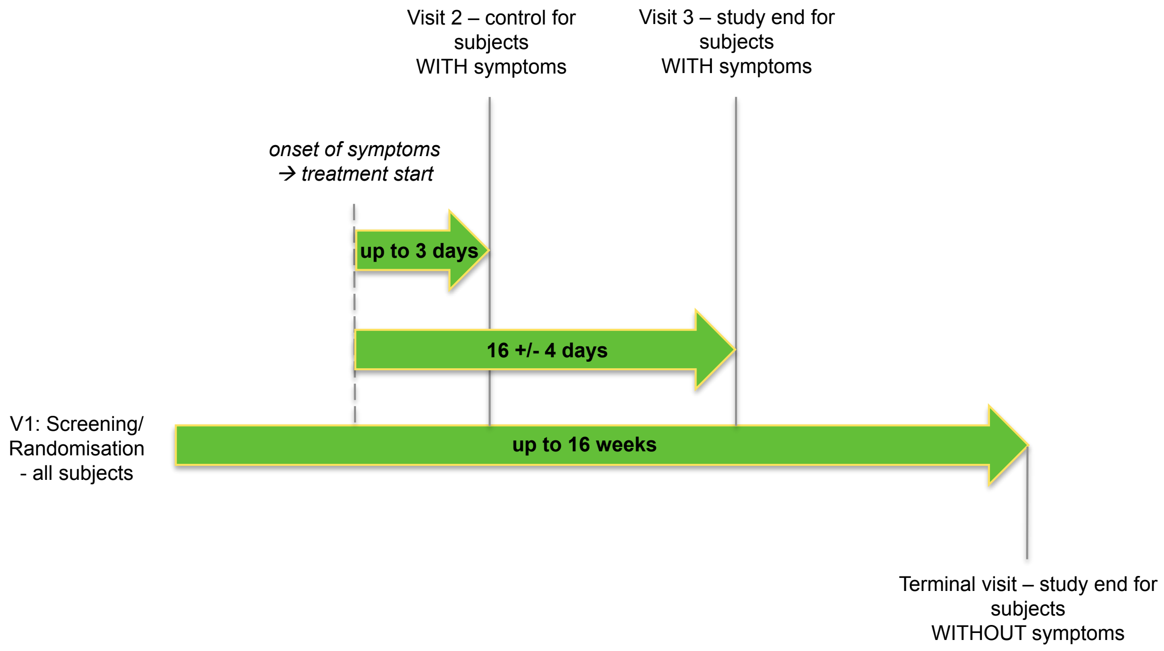

Figure 2: Visit schedule

The study design has been chosen to align with established options for evaluation of common cold (e.g. Barrett et al., 2010; Eccles et al., 2010). The time period of the treatment phase is based on the experience with use of ColdZyme® Mouth Spray (CMS008618) and the typical duration of a common cold episode.

9.2 DURATION OF THE STUDY

The study duration for each subject is maximally 16 weeks ± 7 days (in the case of subjects showing no symptoms). Depending on the advent of a cold illness (symptom onset and consequently of V2) for each subject, an individual's overall study duration will differ.

The expected start date of this study is Q4 2018. The clinical phase of the study is expected to be completed by Q3 2019.

If the required number of subjects with cold symptoms completing V2 and V3 is not achieved in the winter season 2018/2019, recruitment would be stopped in March/April 2019 and then continued in the next winter season (2019/2020).

10 SELECTION OF STUDY SUBJECTS

10.1 NUMBER OF SUBJECTS

A total number of expectedly 600 subjects will be enrolled as eligible study participants and randomised at V1. The first 400 who start using IP upon experiencing symptoms should complete V2 and V3.
Depending on the percentage of subjects from those randomized at V1 who actually delevop symptoms during the study period, the envisaged number of 600 recruited subjects may deviate from the final number of recruited subjects needed.
For reasons of practicability/logistics of recruitment, the targeted number of subjects with symptoms (attending V2 and V3) may be subject to deviations.

10.2 INCLUSION CRITERIA

1. Men and women
2. Age 18 to 70 years old
3. Increased risk for common cold (at least 3 self-reported occurences of common cold within the last 12 months prior to V1) but generally in good health
4. Readiness to comply with trial procedures, including in particular:
   - Use of IP as recommended
   - Filling in diary
   - Keeping habitual life-style, including diet and physical activity level
   - No use of products that may influence the study outcome (e.g. immune suppressants/immune stimulants including natural health products, analgesics/anti-rheumatics, anti-phlogistics, anti-tussives/expectorants, mouth or throat therapeutics, decongestants, antibiotics, anti-histaminergic drugs, nasal drops/spray) during the study (except for the defined “rescue” treatment)
5. Women of child-bearing potential:
   - Have to agree to use appropriate contraception methods
   - Negative pregnancy testing (beta human chorionic gonadotropin test in urine) at V1

Participation is based upon written informed consent by the participant following written and oral information by the investigator regarding nature, purpose, consequences and possible risks of the clinical study.

10.3 CRITERIA FOR CONTINUATION AT V2

Subjects having Visit 2 after onset of common cold symptoms and start of IP use (according to predefined criteria) will continue study participation.

10.4 EXCLUSION CRITERIA

1. Known allergy or hypersensitivity to the components of the investigational product

2. History and/or presence of clinically significant condition/ disorder (self-reported), which per investigator's judgement could interfere with the results of the study or the safety of the subject, e.g.:
   - Nasal disorder (e.g. polyposis, relevant septal deviation, ulcer etc.) and/or reconstructive surgery
   - Asthma, chronic obstructive lung disease or any other acute/chronic air-ways disease/disorder (e.g. chronic cough of any origin)
   - Acute psychiatric disorders
   - Any other acute/chronic serious organ or systemic diseases
3. Influenza vaccination within the last 3 months prior to V1 and during the study
4. Regular use of products that may influence the study outcome (e.g. immune suppressants/immune stimulants including natural health products, analge-sics/anti-rheumatics, anti-phlogistics, anti-tussives/expectorants, mouth or throat therapeutics, decongestants, antibiotics, anti-histaminergic drugs, nasal drops/spray) within the last 4 weeks prior to V1
5. Pregnancy or nursing
6. History of (in the past 12 months prior to V1) or current abuse of drugs, alcohol or medication
7. Participation in the present study of a person living in the same household as the subject
8. Inability to comply with study requirements according to investigator’s judge-ment
9. Participation in another clinical study in the 30 days prior to V1 and during the study

10.5 WOMEN OF CHILD BEARING POTENTIAL

Female subjects may not be pregnant at the time of inclusion into the study. There-fore, pregnancy testing will be performed during screening for women with child bear-ing potential (women of non-childbearing potential are defined as those who either have no uterus, or have undergone ligation of the fallopian tubes, or have permanent cessation of ovarian function due to ovarian failure or surgical removal of the ovaries, or are in the postmenopause as per investigator’s judgement). During the informed consent process, the investigator will advise every woman with child bearing potential that she must use medically recognised contraception during the study duration. In case pregnancy occurs, the subject has to withdraw. Any pregnancy during the study must be reported in writing to the sponsor and the project manager of the CRO by the investigator within 24 hours of becoming aware of the event by sending the re-spective form to:

Fredrik Lindberg, MD, PhD;

email: Fredrik.Lindberg@enzymatica.com

Stephanie Seibt;

email: sk@a-r.com, Fax: +49 30 40008 501

Any initial report must be followed up by a detailed written report.

11 CLINICAL INVESTIGATION TREATMENT

11.1 INVESTIGATIONAL STUDY PRODUCT

CMS008618 is ColdZyme® Mouth Spray, a Class I medical device (CE-marked) under Council Directive 93/42/EEC on Medical Device and MED-DEV Guidance Document 2.4/1. Rev 8, June 2010; with the following composition of the spray solution: glycerol, purified water, cod trypsin, ethanol (<1 %), calcium chloride, trometamol and menthol.

CMS008618 is a non-sterile mouth spray packaged in a primary container consisting of a 20 ml semi-transparent plastic bottle, pump, actuator (spray nozzle) and an actuator terminal cap.

11.2 PLACEBO

The placebo mouth spray solution has the following composition: ethanol (<1 %), menthol and water.

The placebo mouth spray is packaged in a primary container consisting of a 20 ml semi-transparent plastic bottle, pump, actuator (spray nozzle) and an actuator terminal cap.

11.3 DOSAGE AND USE OF IP

The IP (verum and placebo) should be applied every second hour up to 6 times per day with 2 sprays (1 dose) per dose occasion. At V1, the subject will be instructed on the use and the application demonstrated (see section 5.6).

Depending on the timepoint of treatment onset, there may be less IP applied on the first day of use.

The IP use should start when following conditions have been fulfilled:

- answering "Yes" to either of the questions in the subject daily diary: "Do you think/feel you have a cold?" or "Do you think/feel you are coming down with a cold (might be having the first signs of cold)?" AND
- A Jackson score of at least 1 in the subject's cold diary (mild = present, but not disturbing or irritating) for any symptom except headache

The IP should be used until 2 days after the subject is symptom free (=answering "No" to the question "Do you think that you are still sick with this respiratory infection?" for 2 days in a row), but not longer than 10 days in total.

11.4 PRODUCTION, PACKAGING AND LABELLING OF IP

Production and packaging of the IP (verum and placebo), will be taken over by the sponsor, Enzymatica AB. Labelling will be provided by the sponsor (via an independent pharmacist).

The labels will contain the following information (in German), with storage information

only applicable to storage at the subjects's home:

- Randomization code
- Sponsor: Enzymatica AB, Ideon Science Park, 22370 Lund, Sweden
- CRO: analyze & realize GmbH, Waldseeweg 6, 13467 Berlin, Germany, phone: +49 30/40008100
- Name of the investigational product: CMS008618 or placebo
- Batch number:
- Dosage: after symptom start, 2 sprays (1 dose) every second hour up to 6 times per day, on each day after symptom start and for 2 days after symptom stop, but not longer than 10 days in total
- Content per package:
- Study code: 008618
- "For clinical study use only"
- Store:
    - Before first use in a refrigerator
    - After first use at room temperature
- If kept cold until first use, to start use latest by: July 2019
- After first use, must not be used for longer than 10 days
- Keep out of reach of children

11.5 STORAGE, INVENTORY, RETURN, TRACEABILITY AND DOCUMENTATION OF THE INVESTIGATIONAL PRODUCT

The investigational product will be provided to the CRO and/or the investigational sites by the sponsor. The CRO and/or the investigational sites will be responsible for storing the investigational product in a proper place, at the appropriate conditions and inaccessible to unauthorized persons.

The IP should be kept frozen (≤ -18 °C) and cooled transport used for any shipments required until the time point of handing it out to the subjects.

As applicable, the CRO will distribute the investigational product to the investigator who will hand it out to the subjects (alternatively the IP may be shipped directly from the sponsor to the site(s)). The investigator (or designee) will only dispense IP to subjects randomised in this clinical investigation and record the issue and return of the IP.

The IP should be dispensed in a frozen state and the subjects should be provided material and instructions for cooled transport of the IP from the site to their home. They will also be provided with written instructions on handling, storage and use of the IP.

The subjects should store the IP:

- Before first use in a refrigator
- After first use at room temperature. Subjects will be instructed that they should not keep the IP in a place warmer than room temperature and not carry the IP in a pocket of a clothing that may be expected to reach a temperature close to body temperature (e.g. trousers, skirt).

The empty and unused (if any) portion of the IP will be collected from the subjects at the sites and sent to the CRO for the IP accountability (per vial weight). After comple-

tion of the investigation, the unused IP will either be returned to the sponsor or destroyed (as per agreement with the sponsor).

Traceability will be ensured by use of specific batch numbers.

11.6 METHODS OF RANDOMISATION AND BLINDING

The clinical investigation will be conducted in a double-blind randomised manner (block randomisation). The subjects will be randomised at the screening visit. The randomisation list will be provided to the sponsor's assigned responsible person (not involved in the study) by the statistician responsible for generation of the randomisation list. The ratio of randomisation between the verum and placebo will be 1:1. Random numbers will be assigned to the subjects in a sequential order based on time of randomisation at each investigational site (several whole blocks will be allocated to each centre). Randomisation list will be concealed to the investigational sites. It will be stored under lock and key by the sponsor until database closure. After database closure and sign-off of the statistical analysis plan, the sponsor's responsible person will provide the randomisation list to the statistician responsible for the statistical analysis (and the project manager at the CRO, as appropriate).

11.7 BLINDING AND EMERGENCY ENVELOPES

Verum and placebo are identical in appearance, as well as packaging and labelling, so that study participants, CRO and investigators are blinded to treatment assignment. The independent statistician will generate the emergency envelopes for all subjects that will be provided to the CRO and from the CRO to the investigators to be stored in the Investigator Site File (ISF). All emergency envelopes will be collected during close-out of the investigational site at the end of the study.

11.8 UNBLINDING AND PROCEDURES FOR BREAKING CODES

The emergency envelope should be opened by the investigator only in emergency cases (e.g. serious adverse event), in which the investigator suspects a causal relation with the investigational product, requiring unblinding for the decision of the immediate medically required treatment. In case of an emergency unblinding, the investigator will contact the CRO within 24 hours (latest within the very next working day). The CRO will promptly inform the sponsor. Opening of an emergency envelope should be documented in the CRF including the opening person, specific reason for and date and time of unblinding. The same information should be documented on the opened emergency envelope and signed by the investigator. Care must be taken not to unblind any other members of the study team, including CRO and sponsor.

11.9 COMPLIANCE

11.9.1 Overall Compliance to IP use

Evaluation of IP usage will be performed during statistical evaluation at study end, based on the return and documentation of unused investigational product (per vial

weight) as compared to duration of the period defined for IP usage. A subject is classified as compliant when applying at least 80% of the correct quantity of investigational product.

11.9.2 Compliance at the Start of IP use

The IP use should start when the following conditions have been fulfilled:

- answering "Yes" to either of the questions in the subject daily diary: "Do you think/feel you have a cold?" or "Do you think/feel you are coming down with a cold (might be having first signs of cold)?" AND
- Jackson score of at least 1 in the subject's cold diary (mild = present, but not disturbing or irritating) for any symptom except headache

A subject is classified as compliant when applying the IP by the morning of the next day after the first day of a defined common cold (Jackson score ≥ 1, except headache).

11.9.3 Compliance at the Start of Filling out the Subject Cold Diary

The subjects have to start filling out the cold diary on the first day they answer "Yes" to either of the questions in the subject daily diary: "Do you think/feel you have a cold?" or "Do you think/feel you are coming down with a cold (might be having first signs of cold)?".

A subject is classified as compliant when starting records in the cold diary by the morning of the next day after they answer "Yes" to the above questions in the evening.

12 CONCOMITANT TREATMENT

The subjects report use of any concomitant treatment (e.g. other medical devices, medication, any natural health products including food supplements etc.) to the investigator at the first visit. The investigator documents the following information in the CRFs: substance, dosage, start and duration, reason. Subjects will also be advised to inform the investigators of any treatment performed throughout the clinical investigation period.

During the study, the following concomitant treatment listed in the exclusion criteria should not be used:

- products that may influence the study outcome (e.g. immune suppressants/immune stimulants including natural health products, analgesics/anti-rheumatics, anti-phlogistics, anti-tussives/expectorants, mouth or throat therapeutics, decongestants, antibiotics, anti-histaminergic drugs, nasal drops/spray), except for the defined “rescue” treatment
- influenza vaccination.

The following treatment is defined as “rescue” medication during the period of common cold/ start of IP use until V3:

- paracetamol (max. 2 g/day),
- ibuprofen (max. 400 mg/day),
- saline nose drops or nose spray
- as well as antibiotics (in case they are medically required for another ailment following the confirmation of the bacterial infection, however not to be used for common cold).

At enrollment, the investigator informs the subjects that any concomitant treatment that could possibly influence the outcome of the study is not allowed, except for the defined rescue medication.

13 CONDUCT OF THE CLINICAL INVESTIGATION

13.1 VISIT 1 (V1, SCREENING, RANDOMISATION) – ALL SUBJECTS

- Oral and written information about the nature, purpose, possible risks and benefits of the study provided to the subjects by the investigator
- Written consent of the subject to participate; the subject understands the requirements of the clinical investigation and is willing to comply
- Questioning and documentation of the medical history, concurrent diseases and concomitant treatment, demographic and anthropometric data, and physical examination by the investigator
- Verification that the inclusion criteria are met and that there are no violations of the exclusion criteria
- Measurement of blood pressure and pulse rate
- Urinalysis
- Pregnancy test for women with childbearing potential (in urine)
- Issue of subject diary and instructions for record keeping
- Randomisation
- Issuance of IP and instruction for use
- Questioning and documentation of possible occurrence of adverse events (AEs)

Between V1 and V2, the study site will have monthly phone contact with the subjects as a reminder about the study.

13.2 VISITS FOR SUBJECTS WITH SYMPTOMS

13.2.1 Visit 2 (V2), within 1– 3 Days After Symptom Start

- Questioning and documentation of possible occurrence of AEs/device deficiencies
- Questioning and documentation of new or changed concomitant treatment
- Control and re-issue of the subject diary
- Checking criteria for further participation
- Physical examination
- Measurement of blood pressure and pulse rate
- Assessment and documentation of any present symptoms (cold confirmed or another disorder diagnosed)

13.2.2 Visit 3 (V3, Final), 16 ± 4 Days After Symptom Start

- Questioning and documentation of possible occurrence of AEs/device deficien-

cies

- Questioning and documentation of new or changed concomitant treatment
- Return and control of the subject diary
- Return of IP and accountability
- Physical examination
- Measurement of blood pressure and pulse rate
- Global evaluation of efficacy by subjects and investigators (4-point categorical scale)
- Global evaluation of tolerability by subjects and investigators (4-point categorical scale)

13.3 VISIT FOR SUBJECTS WITHOUT SYMPTOMS DURING THE STUDY PERIOD: TERMINATION VISIT (TV), 16 WEEKS ± 7 DAYS AFTER V1

- Questioning and documentation of possible occurrence of AEs
- Questioning and documentation of new or changed concomitant treatment
- Return of the subject diary
- Return of IP

13.4 DEMOGRAPHIC AND ANTHROPOMETRIC DATA

The subject's age (years), gender (male/female) and ethnicity will be assessed and documented in the CRF at V1.

13.5 MEDICAL HISTORY / PHYSICAL EXAMINATION

At V1, the subject will be questioned by the investigator regarding the medical history and will undergo a physical examination. Known pre- or co-morbidities (including the number of self-reported common colds during the past 12 months) as well as any abnormal and/or pathological findings of the examination will be systematically recorded.

A dipstick urinalysis for the assessment of glucose and proteins will be performed for all subjects and a pregnancy test in urine for women with childbearing potential.

13.6 ASSESSMENT OF EFFICACY

13.6.1 Subject Diary

In the daily subject diary, from V1 on, the subject will be asked to answer the questions "Do you think/feel you have a cold?" or "Do you think/feel you are coming down with a cold (might be having first signs of cold)?" (in the morning and in the evening).

From the first day he/she answers „Yes“ to either of the above questions, he/she will have to start filling out the cold diary instead, to:

- record the symptoms on the Jackson scale (twice daily, in the morning and in the evening)
- record the symptoms on the Sore Throat Scale (once daily, in the evening)
- fill out the Wisconsin Upper Respiratory Symptom Survey (WURSS-21) Quality of Life section (once daily, in the evening)
- answer the question "Do you think that you are still sick with this respiratory infection?" (once daily, in the evening)
- record the use of IP (once daily, in the evening)
- record the use of any concomitant treatment / remedies, specifying the time point of use
- if he/she stayed sick at home (away from work, school etc.) due to common cold.

The cold diary should be filled out until 2 days after the subject is symptom free (=answering "No" to the question "Do you think that you are still sick with this respiratory infection?" for 2 days in a row), but not longer than 10 days in total.

If the subjects, in an exceptional case, fail to continue filling out the diary immediately when being symptom free, no data for the 2 subsequent days in the diary would be considered as having no symptoms (corresponding to answering "No" to the question "Do you think that you are still sick with this respiratory infection?").

The total composite Jackson score (Jackson et al., 1958) is calculated by summing the following 8 symptom scores: sore throat, blocked nose, runny nose, cough and sneezing (local symptoms) as well as headache, malaise, and chilliness (systemic symptoms). Local composite Jackson score is derived by summing the local symptoms and systemic composite Jackson score by summing the systemic ones. Symptoms are assessed on a 4-point scale: 0 = none (symptom not present), 1 = mild (present, but not disturbing or irritating), 2 = moderate (symptoms sometimes disturbing/irritating), 3 = severe (symptoms disturbing/irritating most of the time).

The Sore Throat is a 0-10 Likert scale where 0=not sore and 10=very sore (Russo et al., 2013; Shephard et al., 2015).

WURSS-21 (Barrett et al., 2009) is an evaluative illness-specific quality of life instrument with 21 items, designed to assess the negative impact of acute upper respiratory infection, presumed viral (the common cold). In this study, the QoL part of the WURSS-21 will be applied, from item 12 (“think clearly”) to item 20 (“live your personal life”).

Wisconsin Upper Respiratory Symptom Survey – 21 --- *Daily Symptom Report*

Day:	*Date:*	*Time:*	*ID:*

Please fill in one circle for each of the following items:

	Not sick 0	Very mildly 1	2	Mildly 3	4	Moderately 5	6	Severely 7
How sick do you feel **today**?	o	o	o	o	o	o	o	o

Please rate the **average severity of your cold symptoms over the last 24 hours** for each symptom:

	Do not have this symptom 0	Very mild 1	2	Mild 3	4	Moderate 5	6	Severe 7
Runny nose	o	o	o	o	o	o	o	o
Plugged nose	o	o	o	o	o	o	o	o
Sneezing	o	o	o	o	o	o	o	o
Sore throat	o	o	o	o	o	o	o	o
Scratchy throat	o	o	o	o	o	o	o	o
Cough	o	o	o	o	o	o	o	o
Hoarseness	o	o	o	o	o	o	o	o
Head congestion	o	o	o	o	o	o	o	o
Chest congestion	o	o	o	o	o	o	o	o
Feeling tired	o	o	o	o	o	o	o	o

Over the last 24 hours, how much has your cold interfered with your ability to:

	Not at all 0	Very mildly 1	2	Mildly 3	4	Moderately 5	6	Severely 7
Think clearly	o	o	o	o	o	o	o	o
Sleep well	o	o	o	o	o	o	o	o
Breathe easily	o	o	o	o	o	o	o	o
Walk, climb stairs, exercise	o	o	o	o	o	o	o	o
Accomplish daily activities	o	o	o	o	o	o	o	o
Work outside the home	o	o	o	o	o	o	o	o
Work inside the home	o	o	o	o	o	o	o	o
Interact with others	o	o	o	o	o	o	o	o
Live your personal life	o	o	o	o	o	o	o	o

Compared to yesterday, I feel that my cold is…

Very much better	Somewhat better	A little better	The same	A little worse	Somewhat worse	Very much worse
o	o	o	o	o	o	o

WURSS -21© (Wisconsin Upper Respiratory Symptom Survey) 2004
Created by Bruce Barrett MD PhD et al., UW Department of Family Medicine, 777 S. Mills St. Madison, WI 53715, USA

13.6.2 Common cold assessment at V2

Within 1-3 days after start of the symptoms, the subject should attend the Visit 2 at the investigational site. The subjects will be instructed (in the subject diary) to contact the site accordingly.

If the limitation of 3 days may not be held due to e.g. weekend and/or holiday, the Visit 2 could take place on the day following the last non-working day but needs to be clearly documented in the CRF record for Visit 2, together with an explanation to the delayed visit.

During Visit 2, the investigator will perform a physical examination and check the subject diaries.

In some subjects, the initial cold symptoms may possibly not further deteriorate up to a full cold outburst. During the Visit 2, the investigator has to specifically examine whether the cold symptoms initially reported by the subject in the diary are still present and whether any new additional symptoms relevant for the assessment of common cold have appeared.

For (any) observed relevant symptoms, the investigator has to judge whether they are attributable to common cold or if the subject suffers from another ailment (differential diagnosis).

The possible outcome of the investigator's assessments are:

- the subject has a common cold
- the subject suffers from another ailment (e.g. allergic rhinitis, influenza, sinusitis, pharyngitis, tonsillitis, laryngitis, reflux disease, infectious mononucleosis, pneumonia etc.), which needs to be documented as an AE

- the subject has neither a common cold nor any other ailment.

For each common cold, the intensity needs to be assessed and recorded:

- light=does not interfere with the subject's usual function
- moderate=interferes to some extent with the subject's usual function
- severe=interferes significantly with the subject's usual function.

Based on the evaluation of any relevant symptoms by the investigator at V2 (whether they are attributable to common cold), the percentage of subjects with clinically diagnosed common cold will be assessed.

13.6.3 Global Evaluation of Efficacy by the Subjects and Investigators

The subjects and the investigators will evaluate independently the efficacy of the IP by means of a global scaled evaluation with "very good", "good", "moderate" and "poor" at study end.

13.7 ASSESSMENT OF SAFETY

13.7.1 Blood Pressure and Pulse Rate

Sitting blood pressure and pulse rate will be measured using standard products and procedures at all study visits.

13.7.2 Adverse Events

Any AE that occurs during the course of the clinical investigation (for evaluation and reporting see section 14), will be recorded in the CRF. The common cold will not be documented as an AE (being the research object of the study), except in case it is an SAE.

13.7.3 Global Evaluation of Tolerability by the Subjects and Investigators

The subjects and the investigators will evaluate independently the tolerability of the IP by means of a global scaled evaluation with "very good", "good", "moderate" and "poor".

13.8 STUDY DISCONTINUATION

Subjects may withdraw their consent and discontinue their participation in the clinical study at any time, without giving a reason for discontinuation. The investigator may terminate the clinical investigation for single subjects. Further, the sponsor has the right to terminate this clinical investigation.

13.8.1 Withdrawal of Subjects

Reasons for subject's withdrawal may be for example:

- Serious adverse event (SAE)

- Intolerance of the investigational product
- Required additional therapy due to other complaints, which could influence the safety of the subject or the results of the study
- Clinically significant illness or intake of concurrent medication according to exclusion criteria, which could influence the results of the study
- Subject is not compliant with study requirements (according to investigator judgement)
- Withdrawal of informed consent
- Subjects in the study once the number of treated subjects has been completed

Reason, time and specific details of a subject's withdrawal are documented in the CRF. Each subject terminating the study prematurely will be asked to undergo the end-of-study visit, to be documented in the CRF, as follows:

- In case the subject has already completed visit 2, the end-of-study visit would correspond to Visit 3,
- In case the subject has not completed visit 2, the end-of-study visit would correspond to the Termination Visit. Subject not fulfilling criteria for continuation at V2 will complete the TV.

If possible, the IP should be returned by the subject. Subjects who discontinue due to safety reasons should, whenever possible, be seen and assessed by the investigator and be medically advised as appropriate.

13.8.2 Study Discontinuation Criteria

If the clinical investigation has to be discontinued, each subject is to be treated as described above.

The sponsor has the right to terminate this clinical investigation, e.g. for the following reasons:

- Serious, insolvable problems with the quality of the data
- Unforeseeable circumstances at the investigational sites, which require discontinuation of the study (at an individual site and/or in general)
- Unacceptable risks
- New scientific or medical knowledge

14 SAFETY EVALUATION AND REPORTING

14.1 DEFINITIONS

14.1.1 Adverse Event (AE)

Any untoward medical occurrence, unintended disease or injury, or any untoward clinical signs (including an abnormal laboratory finding) in subjects, users or other persons, whether or not related to the investigational medical device.

This includes events related to the investigational device or the comparator and those related to the procedures involved.

For users or other persons this is restricted to events related to the investigational medical device.

14.1.2 Adverse Device Effect (ADE)

Adverse event related to the use of an investigational medical device.

This definition includes adverse events resulting from insufficiencies or inadequacies in the instructions for use, the deployment, the implantation, the installation, the operation, or any malfunction of the investigational medical device.

This includes any event that is a result of a use error or intentional misuse.

14.1.3 Device Deficiency

Inadequacy of a medical device with respect to its identity, quality, durability, reliability, safety or performance, such as malfunction, misuse or use error and inadequate labeling.

14.1.4 Serious Adverse Event (SAE)

Adverse event that,

- led to death,
- led to serious deterioration in the health of the subject, that either resulted in
    - a life-threatening illness or injury, or
    - a permanent impairment of a body structure or a body function, or
    - in-patient or prolonged hospitalization, or
    - medical or surgical intervention to prevent life-threatening illness or injury or permanent impairment to a body structure or a body function,
- led to foetal distress, foetal death or a congenital abnormality or birth defect.

Planned hospitalization for a pre-existing condition, or a procedure required by the clinical investigation plan (CIP), without serious deterioration in health, is not considered a serious adverse event.

14.1.5 Serious Adverse Device Effect (SADE)

Adverse device effect that has resulted in any of the consequences characteristic of a serious adverse event.

14.1.6 Unanticipated Serious Adverse Device Effect (USADE)

Serious adverse device effect, which by its nature, incidence, severity or outcome has not been identified in the current version of the risk analysis report.

Anticipated: an effect, which by its nature, incidence, severity or outcome has been previously identified in the risk analysis report. For relevant precautions applying to CMS008618 (ColdZyme® Mouth Spray), please refer to section 7.2.

14.1.7 Incidents

According to the Ordinance on Medical Devices Vigilance (MPSV), an incident is a malfunction, failure or a modification of the features or performance or an inaccurate label or instruction manual for a medical device, which directly or indirectly caused, may have caused in the past, or may cause in the future, death or a serious aggravation of the state of health of a patient, a user or another person.

14.2 Documenting and Reporting Adverse Events, Adverse Device Effects and Device Deficiencies

14.2.1 Documenting Adverse Events and Adverse Device Effects by the Investigator

During all examinations, the investigator records any observed AEs and those reported by subjects upon questioning.

If an AE occurs, intensity (light=does not interfere with the subject's usual function, moderate=interferes to some extent with the subject's usual function, severe=interferes significantly with the subject's usual function), seriousness (if it is an SAE or not), relationship (not related, unlikely, possible, probable and causal relationship) to the IP, time of occurrence and duration of the observed AE, treatment and resolution/outcome will be recorded in the study documentation. All necessary measures are to be taken to determine the cause of the AE and its possible connection to the study.

14.2.2 Documenting and Reporting Device Deficiencies and Incidents by the Investigator

During all examinations after applying the investigational product, the subjects will be asked about any device deficiencies observed. If a device deficiency is reported, the deficiency including the assessment if it may be regarded as an incident will be recorded in the study documentation.

Reportable are all device deficiencies that might have led to a serious adverse event if

a) suitable action had not been taken, or

b) intervention had not been made or

c) if circumstances had been less fortunate,

as well as any incidents.

The reporting is handled under the SAE reporting system and will be reported as specified in the form "Report on device deficiencies" ("*Bericht über Mangel des Medizinproduktes / Vorkommnis*", in the ISF) immediately after becoming aware (not later than 3 calendar days) to the sponsor and the CRO.

Contacts:

Fredrik Lindberg, MD, PhD;

email: Fredrik.Lindberg@enzymatica.com

Stephanie Seibt;

email: sk@a-r.com, Fax: +49 30 40008 501

The initial reports will be followed up until event resolution, or for 14 calendar days after end-of-study/subject withdrawal, whichever comes first.

14.2.3 Reporting Serious Adverse Events by the Investigator

All serious adverse events that occur for any reason during the study, will, even when the cause is not connected to the use of the device, be reported by the investigator as specified in the form "Report on serious adverse event" ("*Bericht über schwerwiegende unerwünschte Ereignisse*", in the ISF) within 24 hours of awareness to the sponsor and the CRO.

Contacts:

Fredrik Lindberg, MD, PhD;

email: Fredrik.Lindberg@enzymatica.com

Stephanie Seibt;

email: sk@a-r.com, Fax: +49 30 40008 501

The initial SAE reports will be followed up until event resolution, or for 14 calendar days after study close-out/subject withdrawal, whichever comes first.

14.2.4 Safety Evaluation and Reporting by the Sponsor

The sponsor is responsible for the ongoing safety evaluation of the clinical investigation, reviewing of the device deficiencies, incidents and SAEs reported by the investigator and any further reporting to the Ethics Committee (EC) and the Competent Authority as appropriate

15 STATISTICS

The present clinical investigation is designed as a double-blind, randomized, parallel-group, placebo-controlled study to test the study hypothesis that treatment with CMS008618 is superior to placebo in the efficacy treatment of naturally occurring common cold.
The primary objective of the present clinical trial is to evaluate the illness-specific quality of life as assessed per AUC of the WURSS-21 QoL composite subscore during first 8 days of symptoms.

The statistical analyses will be confirmatory for the primary endpoint and the major secondary endpoints, and exploratory for all other secondary endpoints. All statistical testing will performed two-sided. The confirmatory significance level is fixed to a type I error rate alpha of 5% (two-sided).

Statistical analysis will be done by the sponsor or under the authority of the sponsor. A general description of the statistical methods to be used to analyze the efficacy and safety data is outlined below. Specific details will be provided in the Statistical Analysis Plan (SAP).

15.1 STATISTICAL HYPOTHESES

A lower value of the primary endpoint (i.e., AUC of WURSS-21 QoL composite subscore assessed during first 8 days of symptoms) corresponds to fewer complaints, a higher value corresponds to more complaints. The study is aiming to show superiority of CMS008618 compared to placebo, i.e. aiming at lower values of the AUC values in the verum group compared to the placebo group.

The null hypothesis H_0 and the alternative hypothesis H_A for the confirmatory analysis of the primary endpoint are formulated as follows:

- H_0: $\mu_{verum} = \mu_{placebo}$, i.e. no difference between verum and placebo with respect to the primary endpoint.
- H_A: $\mu_{verum} \neq \mu_{placebo}$, i.e. difference between verum and placebo with respect to the primary endpoint.

The null hypotheses H_0 and the alternative hypotheses H_A for the major secondary endpoints are formulated analogously.

15.2 SIGNIFICANCE LEVEL

The confirmatory type I error rate for the study is set to 0.05 (two-sided). Due to the testing of a-priori ordered hypotheses, no multiplicity correction is considered necessary (Points to consider on multiplicity issues in clinical trials, CPMP/EWP/908/99, 2002).

15.3 SAMPLE SIZE CALCULATION

The sample size for the present study is calculated so that the comparison of the verum treatment group and the placebo treatment group for the primary endpoint has 90% power to detect a relevant clinical effect size δ (i.e., the ratio of the absolute val-

ue of the difference between two means and the standard deviation) with a two-sided significance level α=0.05 as outlined below.

Based on the results of a previously conducted trial with the same IP in a comparable setting (sponsor's data on file), the difference between the verum group and the placebo group regarding the mean AUC of the WURSS-21 QoL composite subscore during first 8 days of symptoms is expected to be at least 33.900. The associated common standard deviation σ is expected to be 92.700. Therefore, the relevant clinical effect size $\delta = |\mu_{verum} - \mu_{placebo}| / \sigma$ (i.e. the ratio of the difference between verum and placebo and the standard deviation) which should be detected with adequate power is assumed to be 0.366. This corresponds to $p_1 = P(X<Y) = 0.602$ which is the probability that an observation, X, in group 1 will be less than an observation Y in group 2 when the alternative hypothesis is true. The null hypothesis being tested is that $p_1 = 0.5$.

Under these assumptions, a sample size of n = 169 subjects in each treatment group will have 90% power to detect a probability of 0.602 that an observation in the verum group is less than an observation in the placebo group using a Wilcoxon (Mann-Whitney) rank-sum test with a 0.05 two-sided significance level (nQuery Advisor® Release 7.0).
Based on the sample size in the study and taking into account an assumption for a power of 80%, an effect size of 0.315 with regard to the main secondary endpoints (Jackson score AUC and exposure to relevant concomitant medication) would be detectable (with a power of 80% and a sample size of n = 169 subjects in each treatment group, p1 = P(X<Y) = 0.588 is calculated, corresponding to an effect size d = 0.315.)
Thus, considering the planned 1:1 randomization with two treatment groups, n = 338 subjects (with n = 169 subjects per treatment group) are required for the primary confirmatory statistical analysis of the study.
Of all subjects originally randomized in a 1:1 manner, only those having the specified symptoms (see section 11.3) will actually start using the IP. Therefore, it is rather probable that the ratio of subjects actually having used IP and completing the study may differ from the original 1:1 ratio. In order to ensure the minimal number of required subjects per study group, 18% of additional subjects completing V2 and V3 are envisaged, resulting in the total number of 400 treated subjects.

15.4 DROP-OUT RATE

Drop-outs after V2 will be replaced until the required number of at least 400 subjects completing V2 and V3 is reached.

15.5 ANALYSIS SETS

The statistical evaluation will be based on separate, hierarchically organized analysis sets as defined below:

- All subjects randomized set

  All subjects which were randomized to the treatment groups.

- All subjects treated set

All subjects of the all subjects treated set which received at least 1 dose of the IP.

- Full analysis set

  All subjects in the all subjects treated set for which the primary endpoint is available. Subjects which have not been enrolled in the study according to the inclusion/exclusion criteria, but this had only been detected during the study or during the process of data cleaning will be excluded from the full analysis set but remain in the all subjects treated set (ICH E9, 1998)

- Per-protocol analysis set

  All subjects of the full analysis set without any major violation of the protocol and its procedures. Major violations will be defined during the blind data review (see 15.6).

A flowchart will show the final disposition of all subjects in the different analysis sets according to the treatment groups together with a summary of the reasons for exclusion. Demographic and baseline characteristics will be analyzed for all four analysis sets. Analyses with respect to safety will be performed for the all subjects treated set. Analyses with respect to efficacy will be conducted both for the full-analysis set and the per-protocol analysis set. The data of subjects who were not randomized will be listed and documented at the site and stored in the ISF and TMF. They will not be entered in the clinical database or otherwise reported.

All subjects randomized will receive the IP, but will only start using it after onset of common cold symptoms. The decision of whether or not to begin treatment could not be influenced by knowledge of the assigned treatment, as the study is performed in a double-blind manner.

15.6 ELIGIBILITY, PROTOCOL DEVIATIONS

A detailed description of the procedures and criteria used for data review and sort out will be provided prior to start of the analysis and before unblinding of the treatment allocation.
Each subject's allocation to the different analysis sets will be identified and mutually agreed upon during data review prior to unblinding of the treatment allocation. Deviations from the protocol including violations of inclusion/exclusion criteria will be assessed as 'minor' or 'major' based on the conjoint decisions according to the specifications outlined in the SAP or a separate document.
Relevant deviations from the protocol will lead to the exclusion of a subject from the per-protocol analysis set. Listings will be prepared to show the eligibility of all subjects for the different analysis sets and a summary will be given on the number of subjects per analysis set.

15.7 DEMOGRAPHICS AND OTHER BASELINE CHARACTERISTICS

Demographics and other baseline characteristics will be summarized by means of descriptive statistics for continuous data and frequency tables for categorical data. Summary tables will be provided for all analysis sets.

15.8 ASSESSMENT OF COMPLIANCE

Evaluation of overall compliance with respect to IP use will be based on the return and documentation of unused IP (per vial weight) as compared to duration of the period defined for IP usage (see chapter 11.9.1). Assessment of compliance to start of IP use and to start of filling out the subject cold diary is described in chapters 11.9.2 and 11.9.3. Compliance data will be summarized by means of descriptive statistics for continuous data and frequency tables for categorical data for all analysis sets besides the all subjects randomized set.

15.9 ANALYSIS OF PRIMARY ENDPOINT

The primary endpoint is defined as the AUC of WURSS-21 QoL composite subscore assessed during first 8 days of symptoms.

The WURSS-21 QoL composite subscore is calculated by summing the scores of the 9 consecutive items of WURSS-21, from item 12 ("think clearly") to item 20 ("live your personal life"), as documented in the subject diary (see 13.6.1). The AUC will be assessed by applying the trapezoidal approximation.

The confirmatory analysis of the primary endpoint will be performed for the full analysis set. Exploratory analyses will be performed for the per-protocol analysis set.

Data of the primary endpoint will be summarized by descriptive statistics using appropriate tabulation.

The two-sided confirmatory null-hypothesis will be tested with the non-parametric two-sided Wilcoxon (Mann-Whitney) rank-sum test.

15.10 ANALYSIS OF MAJOR SECONDARY ENDPOINTS

The major secondary endpoints are ranked according to clinical relevance as:

1. AUC days 1-8 composite daily severity of all symptoms within the Jackson score (mean of morning and evening) (day 1 is the first day of symptom recording). The two-sided confirmatory null-hypothesis will be tested with the non-parametric two-sided Wilcoxon (Mann-Whitney) rank-sum test.
2. Exposure to any concomitant treatment (including natural health products) that may affect common cold symptoms - immune suppressants/immune stimulants, analgesics/anti-rheumatics, anti-phlogistics, antitussives/expectorants, mouth or throat therapeutics, decongestants, antibiotics, anti-histaminergic drugs, nasal drops/spray or any medication/treatment known to affect common cold symptoms - at any dose, expressed as number of days with concomitant treatment during the first 4 days for each subject (based on diary data). The two-sided confirmatory null-hypothesis for the second major secondary endpoint will be tested using the non-parametric two-sided Wilcoxon (Mann-Whitney) rank-sum test.

In order to control the overall type I error rate, the primary endpoint and the two major secondary endpoints will be tested in a fixed sequence as a-priori ordered hypotheses. That is, the first major secondary endpoint will be tested only if the primary endpoint is positive (ie, $p < 0.05$), and the second major secondary endpoint will be tested

only if the first major secondary endpoint is positive. No confirmatory claims will be based on endpoints that have a rank lower than or equal to that variable whose null hypothesis is the first that could not be rejected.

The confirmatory analysis of the major secondary endpoints will be performed for the full analysis set. Exploratory analyses will be performed for the per-protocol analysis set.

Data of the major secondary endpoints will be summarized by descriptive statistics using appropriate tabulation.

15.11 ANALYSIS OF OTHER SECONDARY ENDPOINTS

Analyses of other secondary endpoints will be performed primarily for the full analysis set and for selected parameters also for the per-protocol analysis set. All other secondary endpoints will be analyzed exploratory only and will be descriptively assessed. For continuous data, standard statistical characteristics will be presented (number of subjects with non-missing data, mean, standard deviation, median, minimum and maximum and quartiles). The values of continuous data may also be transformed into categorical ordinal classes according to clinical criteria to determine their frequency distribution. Categorical data (nominal or ordinal) will be summarized using frequency tables.

Continuous other secondary endpoints will be analyzed for between-group comparison using the non-parametric two-sided Mann-Whitney-U-test. Multiple testing will be performed without exploratory adjustment for multiple testing. For categorical other secondary endpoints chi² tests will be used for between-group comparison.

A more detailed description of the planned descriptive and exploratory analyses will be presented in the SAP.

15.12 ANALYSIS OF SAFETY ENDPOINTS

All subjects treated at least once with the IP will be used to evaluate issues of tolerability and safety. Safety evaluations will be based on assessment of adverse events, the global evaluation of tolerability by subject and investigator, physical examination, vital signs, and assessment of adverse device effects and reporting of device deficiencies.

<u>Adverse Events</u>
All reported adverse events with onset during the treatment phase (i.e., treatment-emergent adverse events) will be included in the analysis. For each adverse event, the number and percentage of subjects who experience at least 1 occurrence of the given event will be summarized (also including the break down by intensity and relationship to the IP).
Tabulated summaries or listings may be provided, as appropriate, for those subjects who discontinue treatment due to an adverse event, or who experience a severe or a serious adverse event.

<u>Global Assessment of Tolerability</u>
Global assessment of tolerability of the IP by the subject and investigator will be presented by frequency tabulation providing n (%) of subjects.

<u>Physical Examination</u>

Physical examination findings will be summarized at each scheduled time using frequency tabulations.

Vital Signs
Descriptive statistics of blood pressure and of pulse rate measurements as well as changes from baseline will be summarized at each scheduled time.

Device Deficiencies

The number and percentage of subjects with a given device deficiency will be displayed in a frequency table. Details on abnormal findings will be displayed in individual subject data listings. Device deficiences which cause an adverse event will be included in the adverse event evaluation.

15.13 INTERIM ANALYSIS

No interim analysis is planned in this study.

15.14 SUBGROUP AND SENSITIVITY ANALYSIS

Subgroup analyses may be defined in the SAP after a blinded review of the data, before unblinding of the data.

Exploratory sensitivity analyses of the time course of the WURSS-21 QoL composite subscore assessed during first 8 days of symptoms will be done by using a multivariate nonparametric model of longitudinal data in a two-factorial design with the fixed effect factor 'treatment' and the repeated measures factor 'study day', testing for treatment differences, impact of study day and the interaction 'treatment x study day' (Brunner et al., 2002). In addition, a multivariate nonparametric covariance analysis of longitudinal data in a two-factorial design as decribed above with the baseline value of the WURSS-21 QoL composite subscore as covariate will be performed (Bathke & Brunner, 2003).

A more detailed description of the planned exploratory sensitivity analyses will be presented in the SAP.

15.15 MISSING DATA

All available data will be included in the analyses and will be summarized as far as possible. A detailed description of the handling of missing data will be provided in the SAP after blinded data review, before unblinding of the data. A sensitivity analysis may be performed to evaluate the influence of missing values.

15.16 STATISTICAL ANALYSIS PLAN

Before database closure, a statistical analysis plan (SAP) will be provided, presenting planned statistical analyses as well as any possible deviations or additions of the data analyses originally defined in the CIP before clinical study start. Changes in the planned statistical methods and analyses after approval of the SAP will be documented in the clinical study report. The results of the statistical analyses of the study will be documented in the statistical report. The statistical report will provide the basis for the preparation of the study report.

16 ETHICAL AND LEGAL CONSIDERATIONS

16.1 REGULATORY ASPECTS

This clinical investigation will be performed based on the principles of the World Medical Association (Declaration of Helsinki, version applicable for the involved investigators), ICH GCP E6 (R2), German Act on Medical Devices (MPG) §23b and ISO 14155. Further, for reporting of deficiencies, MEDDEV 2.7/3 rev. 3 and for reporting of incidents, the Ordinance on Medical Devices Vigilance and MEDDEV 2.12/1 rev. 8 Vigilance will be followed.

16.2 INDEPENDENT ETHICS COMMITTEE

This clinical investigation will be evaluated by an EC before inclusion of the first subject. The CRO will submit the CIP and relevant study documents to the EC to request for opinion.

16.3 SUBJECT INFORMATION AND INFORMED CONSENT FORM

The subject must declare informed consent before participation in the clinical investigation, i.e. before any study specific procedure is performed.

The investigator will ensure that the subject is given full and adequate oral and written information (subject information) about the nature, purpose, consequences and possible risk of the clinical investigation. Subjects must be informed that they are free to withdraw from the study at any time without any resulting disadvantages, how personal and health-related data will be collected and used during the study, and that their identity and medical information will not be disclosed. The subject should be given the opportunity to ask questions and should be allowed sufficient time to consider the information provided. The subject’s signed and dated informed consent must be obtained before conducting any study specific procedure. The investigator(s) must store the original, signed and dated informed consent forms in the ISF. A copy of the signed and dated informed consent form must be given to the subject, along with a copy of the insurance conditions.

If new information becomes available that can significantly affect a subject’s future health and medical care, that information will be provided to the subject(s) affected in written form. If relevant, all affected subjects will be asked to confirm their continuing informed consent in writing.

16.4 INVESTIGATOR’S OBLIGATIONS

By signing this document, the investigator confirms to adhere to the CIP and all applicable national and international regulations and guidelines. The investigator has to ensure that all other study (sub-) investigators and study personnel assisting in the present clinical investigation are qualified by training for their individual responsibilities and functions and informed about the clinical study documents, about the IP, and the investigational procedures.

A delegation log will be set up and updated during the course of the study, in which the investigator will list all study team members including their delegated responsibilities.

The investigators will permit access to source data for study-related monitoring as well as in case of audits and inspections.

16.5 AMENDMENTS TO THE CIP

All amendments to the CIP need agreement between the sponsor and the coordinating investigator. Every substantial amendment to the CIP or the subject information including the informed consent form needs to be reviewed by the EC prior to implementation.

Under emergency circumstances, deviations from the CIP to protect the rights, safety and well-being of human subjects may proceed without prior approval of the sponsor and the EC.

16.6 DATA PROTECTION AND CONFIDENTIALITY

The CRO and the study sponsor's representative will ensure the right of the study participants to protection from invasion of privacy. The investigators have to comply with the data protection laws of the Federal Republic of Germany ("Bundesdatenschutzgesetz").

Following consent to participation in the clinical investigation, the subject will be assigned a screening number. Subjects meeting the eligibility criteria will be assigned a random number. The collected data will be made available to the CRO and the study's sponsor only in pseudonymous form, to minimize the chances of matching the data to an individual person. Only the age and gender will be recorded in CRF (no initials, no date of birth). The paper based identification list will be confidentially retained by the investigator.

In case of SAE related documentation provided to the CRO and the study sponsor, the investigator will ensure the pseudonymity of the copies of the documentation provided.

With the receipt of the CIP, the investigator is bound to treat all information contained herein as strictly confidential. He/she is further required to inform his support staff or other personnel that may have access to the CIP of this confidentiality.

17 QUALITY CONTROL AND QUALITY ASSURANCE

Quality assurance and quality control systems will comprise e.g.:

- Training
- Monitoring visits
- Double data entry
- SOPs of the CRO

17.1 TRAINING

The investigator and the study monitor must make sure that study staff has been appropriately trained and has received the relevant information relating to this clinical investigation.

17.2 MONITORING

The purpose of monitoring is to verify that the conduct of the clinical investigation complies with the approved CIP, subsequent amendments and applicable regulations. Before the initiation of the clinical investigation, a monitor will verify the adequacy of the investigational site and facilities and discuss with the investigator(s) and other personnel involved in the clinical investigation their responsibilities with regard to the clinical investigation. At the end of the clinical investigation a close-out visit will be performed at the investigational site.

During the clinical investigation, a monitor will have regular contacts with the investigational site, including visits to verify that all data in the CRFs are complete, recorded in a timely manner and consistent with source data, that signed and dated informed consent forms have been obtained from each subject at the time of enrollment and before any clinical study-related procedures are undertaken, that the clinical investigation is being performed according to the CIP, and in accordance with ICH GCP (E6), ISO14155 and applicable regulations.

Further details with respect to monitoring including the extent of source data verification will be laid out in a Monitoring Plan.

17.3 CIP DEVIATIONS

No systematic or prospective deviations from the CIP are tolerated. CIP deviations will be evaluated during blind data review by the sponsor, the CRO, and the biometrician in order to define the study data sets.

17.4 AUDITS AND INSPECTIONS

Authorised representatives of the sponsor and / or regulatory authorities, if applicable, may visit the study centres to perform audits or inspections, including source data verification. The purpose of an audit or inspection is to systematically and independently examine all intervention-related activities and documents to determine whether these activities were conducted, and whether data were recorded, analysed and accurately reported according to the CIP, ICH GCP, ISO 14155 and applicable regulations.

18 DATA HANDLING AND RECORD KEEPING

18.1 SOURCE DATA

All required source data will be recorded in the subject file and subsequently transcribed into CRF. The source data entries must allow to assess whether the study including all procedures was conducted according to CIP. The study diaries and questionnaires act both as source data as well as a part of the CRF.

18.2 DATA DOCUMENTATION

The CRO will provide the investigator with a CRF for each subject. In this CRF, the investigator (or delegate) will in a pseudonymised manner enter all ascertained findings concerning the subject which are documented in the source data. Only the screening and randomization numbers will designate the subject in the CRF.

It is the investigator's responsibility to maintain adequate and accurate CRF to record all observations and other data pertinent to the study. The CRF must be completed with a ballpoint pen, in a correct and legible manner and must be kept current at all times. In no case may corrections be erased or concealed. If a correction should be necessary, the incorrect entry is to be left with a single strike through. The correction is to include the date and signature of the authorized person making the correction.

The investigator confirms by signature the accuracy of all entries in the CRF. The CRF will be checked by the monitor according to the monitoring plan. The investigator should keep subject records separate from the CRF at the investigational site.

18.3 INVESTIGATOR SITE FILE

The investigator will receive a document folder containing the obligations pertaining to him/her during the clinical investigation. It is mainly composed of:

- CIP (and amendments, if applicable)
- Subject information and informed consent
- Ethics committee documents
- CRFs (incl. diaries/questionnaires)
- Form for serious adverse events and report on device deficiencies
- Study initiation monitoring report
- Monitoring log
- Investigational product accountability documentation
- Relevant applicable regulations
- Screening list
- Subject identification list
- Site signature and delegation sheet

- Clinical investigation insurance documentation

18.4 DATA MANAGEMENT

All collected data will be documented in the CRF and checked by the monitor at the investigational site at regular intervals for plausibility and completeness according to the agreed extent. The completed CRFs will be returned to the CRO for data entry.

Following double data entry into the study database, any inconsistencies detected will be resolved during the data cleaning process by using data clarification forms (queries). The investigational site has to either confirm that the queried data is correct, or alternatively provide the correct data. A copy of the data clarification form will be filed with the study documents at the site. Once the database is declared to be complete and accurate, it will be locked.

18.5 ARCHIVING

Source data are a component of the subject records and remain at the investigational site. The completed CRFs will be returned to the CRO for data entry and archived along with other study documentation and correspondence concerning the study (Trial Master File) by the sponsor. The ISF including copies of CRFs will remain at the investigational site. The documents will be retained at least 10 years after the end of the clinical investigation.

18.6 PRESENTATION OF DATA – FINAL REPORT

At the end of the clinical investigation, a study report in alignment with the ISO 14155 will be generated.

19 FINANCING / INSURANCE

The financial sponsor of this clinical investigation is Enzymatica AB. For the event of an injury arising from participation in the clinical investigation, all subjects are covered by a clinical investigation insurance.

20 PUBLICATION POLICY

The results of this clinical investigation may be published with the written agreement of the study sponsor.

21 LITERATURE

Clarsund M, Fornbacke M, Uller L, Johnston SL, Emanuelsson CA. (2017) A Randomized, Double-Blind, Placebo-Controlled Pilot Clinical Study on ColdZyme® Mouth Spray against Rhinovirus-Induced Common Cold. Open Journal of Respiratory Diseases, 7, 125-135.

Barrett B, Brown R, Rakel D, Mundt M, Bone K, Barlow S, Ewers T. Echinacea for treating the common cold: a randomized trial. Ann Intern Med. 2010 Dec 21;153(12):769-77.

Bathke A & Brunner E. (2003). A Nonparametric Alternative to Analysis of Covariance. Recent Advances and Trends in Nonparametric Statistics. (Akritas MG & Politis DN), Amsterdam: Elsevier B.V., 109-120.

Brunner E, Domhof S & Langer F (2002). Nonparametric Analysis of Longitudinal Data in Factorial Experiments. Wiley & Sons, New York

Committee for proprietary medicinal products (CPMP). Points to consider on multiplicity issues in clinical trials. CPMP/EWP/908/99. 19 Sept 2002.

Eccles R. Understanding the symptoms of the common cold and influenza. Lancet Infect Dis. 2005 Nov;5(11):718-25.

Eccles R, Meier C, Jawad M, Weinmuellner R, Grassauer A, Prieschl-Grassauer E. Efficacy and safety of an antiviral Iota-Carrageenan nasal spray: a randomized, double-blind, placebo-controlled exploratory study in volunteers with early symptoms of the common cold. Respir Res. 2010 Aug 10;11:108.

Eccles R. Is the common cold a clinical entity or a cultural concept? Rhinology. 2013 Mar;51(1):3-8.

Heikkinen T, Jarvinen A. The common cold. Lancet 2003; 361: 51–59.

Hendley, JO. The host response, not the virus, causes the symptoms of the common cold. Clinical Infectious Diseases, vol. 26, no. 4, pp. 847–848, 1998.

ICH Harmonised Tripartite Guideline, Statistical Principles For Clinical Trials E9, Current Step 4 version, 5 February 1998.

Investigator's Brochure, 2018.

Jackson GG, Dowling HF, Spiesman IG, Boand AV. Transmission of the common cold to volunteers un-der controlled conditions. Arch Intern Med 1958, 101:267-278.

Johnston, SL. Problems and prospects of developing effective therapy for common cold viruses. Trends in Microbiology, vol. 5, no. 2, pp. 58–63, 1997.

Russo M, Bloch M, de Looze F, Morris C, Shephard A. Flurbiprofen microgranules for relief of sore throat: a randomised, double-blind trial. Br J Gen Pract. 2013 Feb; 63(607): e149–e155.

Shephard A, Shea T, Smith G, Schachtel E, Schachtel B. An alternative to antibiotics for the symptomatic relief of sore throat – evidence from a randomised controlled study of flurbiprofen lozenge. 25th European Congress of Clinical Microbiology and Infectious Diseases (ECCMID), 25–28 April 2015, Copenhagen, Denmark.

Stefansson B, Gudmundsdottir A, Clarsund M. A medical device forming a protective barrier that deactivates four major common cold viruses, Virol Res Rev, 2017 doi: 10.15761/VRR.1000130 Volume 1(5): 1-3

Tyrrell DAJ. Erkaeltungskrankheit – ein Lehrbuch für die Praxis. G.-Fischer-Verlag, Stuttgart-Jena-New York, 1996.

Wagner H. Pflanzliche Immunstimulanzien. Zur Prophylaxe und Therapie von Erkaeltungskrankheiten. Z Phytother 17 (1996), 79-85.

Witek TJ, Ramsey DL, Carr AN, Riker DK. 2015, “The Natural History of Community-Acquired Common Colds Symptoms Assessed Over 4-Years”, Rhinology, vol. 53, no. 1, 81-88.

22 SIGNATURES

I declare myself in agreement with this CIP and its appendices.

Study sponsor's representative
Project manager: 28MAY2019, Ida Nelson

Date, Ida Nelson

Coordinating investigator/
Medical expert CRO: 31. 5. 2019 [illegible]

Date, Prof. Ralf Uebelhack, MD

Head of Scientific Affairs CRO: 31-MAY-2019 [illegible]

Date, Gordana Bothe, PhD

Project manager CRO: 29. May 2019 Stephanie Seibt

Date, Stephanie Seibt

Biometrician: 29MAY2019 [illegible]

Date, Werner Baurecht

22.1 STUDY ADMINISTRATIVE STRUCTURE

Study sponsor
Ida Nelson
Enzymatica AB
Ideon Science Park, 223 70 Lund, Sweden
Tel: +46 46 286 31 00
email: ida.nelson@enzymatica.com

Project manager CRO
Stephanie Seibt
analyze & realize GmbH
Waldseeweg 6, 13467 Berlin, Germany
Tel: +49 30 40008 144
Fax: +49 30 40008 501
email: sseibt@a-r.com

Coordinating investigator/Medical expert CRO
Prof. Ralf Uebelhack, MD
analyze & realize GmbH
Weissenseerweg 111, 10369 Berlin, Germany
Tel: +49 30 40008 105
Fax: +49 30 40008 501
email: ruebelhack@a-r.com

Study coordination CRO
Sandra Weiss
analyze & realize GmbH
Waldseeweg 6, 13467 Berlin, Germany
Tel: +49 30 40008 201
Fax: +49 30 40008 501
email: sweiss@a-r.com

Monitoring lead CRO
Yvette Röske, PhD
analyze & realize GmbH
Waldseeweg 6, 13467 Berlin, Germany
Tel: +49 30 40008 104
Fax: +49 30 40008 501
email: yroeske@a-r.com

Biometry lead
Werner Baurecht
acromion GmbH
Europaallee 27-2, 50226 Frechen, Germany
Tel: +49 2234 2037 37-17
Fax: +49 2234 2037 37-9
Email: werner.baurecht@acromion-gmbh.com

22.2 STUDY FLOW CHART

22.2.1 Subjects with symptoms

**Procedure/ Assessment**	**Visit 1** **Screening / Randomisa-tion**	**Visit 2** within 1–3 days after symptom start	**Visit 3** **Final visit** 16 ± 4 days after symptom start
Subject information	X		
Written informed consent	X		
Anamnestic, demographic, anthropometric data	X		
Inclusion and exclusion criteria	X		
Medical history/ concurrent diseases	X		
Concurrent treatment	X	X	X
Physical examination	X	X	X
Blood pressure and pulse rate	X	X	X
Documentation of symptoms (cold/other disease)		X	
Checking criteria of further partic-ipation		X	
Urinalysis	X		
Pregnancy test for women of childbearing potential (in urine)	X		
Issue of subject diary and instruc-tion	X		
Check and re-issue of subject diary		X	
Return/control of subject diary			X
Randomisation	X		
Issue of IP and instruction	X		
Collection of IP and accountability			X
Adverse events	X	X	X
Device deficiencies		X	X
Global evaluation of efficacy and tolerability by subject and investi-gator			X

22.2.2 Subjects without symptoms

Procedure/ Assessment	Visit 1 Screening / Randomisa-tion	Terminal visit 16 weeks ± 7 days after V1
Subject information	X	
Written informed consent	X	
Anamnestic, demographic, anthropometric data	X	
Inclusion and exclusion criteria	X	
Medical history/ concurrent diseases	X	
Concurrent treatment	X	X
Physical examination	X	
Blood pressure and pulse rate	X	
Documentation of symptoms (cold/other disease)		
Checking criteria of further partic-ipation		
Urinalysis	X	
Pregnancy test for women of childbearing potential (in urine)	X	
Issue of subject diary and instruc-tion	X	
Check and re-issue of subject diary		
Return of subject diary		X
Randomisation	X	
Issue of IP and instruction	X	
Collection of IP		X
Adverse events	X	X
Device deficiencies		
Global evaluation of efficacy and tolerability by subject and investi-gator		
