# Supplementary material for: Expression of Concern: Early intervention with ColdZyme mouth spray after self-diagnosis of common cold: A randomized, double-blind, placebo-controlled study
Source: PLoS One. 2026 Jun 16;21(6):e0351708. doi: 10.1371/journal.pone.0351708 (PMC13271471; doi:10.1371/journal.pone.0351708)
Supplement: S1 Data — (PDF) [file pone.0351708.s001.pdf]

Data Dictionary and Variable Mapping for Individual-Level Raw Data

Study CMS008618

1. File Description

This Supplementary File provides a **data dictionary and variable-level mapping** for the **raw, unprocessed individual-level data** from study CMS008618 that underlie all tables and figures reported in the manuscript and in *S1_File Data in manuscript*.

All variables originate from the validated electronic Case Report Form (eCRF) system used in the study and are provided at **subject-level and visit/day-level**, without aggregation. This file enables **independent verification and reproduction** of all reported summary statistics, in accordance with the PLOS ONE Data Availability Policy.

2. Subject Identification and Data Structure

Each subject is uniquely identified using a combined identifier derived from site and subject number. These identifiers are present on all records and allow linkage across datasets.

**Variable**	**Description**
SITEID	Study site identifier
SUBJID	Subject number within site
H_SUBJID	Unique subject identifier (SITEID–SUBJID)
VISITCODE	Visit identifier (e.g. V01, V02, V03)
VISITDATE	Date of visit or diary entry

3. Demographic Variables (Table 1)

The following raw variables underlie all demographic summaries presented in Table 1.

**Variable**	**Description**
GENDER	Sex (male/female)
R_AGE	Age at baseline (years)

RACE	Ethnic category
RACEOTH	Free-text ethnicity (if applicable)

4. Diary-Based Symptom Severity Data (Jackson Score)

These variables capture **daily symptom severity** and are used to derive total Jackson scores, AUCs, durations, and frequency-based endpoints (Tables C.4.3, C.4.19, C.4.20).

4.1 Morning Diary (QS2)

Variable	Description
QS2DT	Diary date
QS2DAYS	Study day number
QS2_F1–QS2_F8	Individual Jackson symptom severity items (ordinal scale)

4.2 Evening Diary (QS4)

Variable	Description
QS4_F1–QS4_F8	Evening symptom severity items
QS4_F9–QS4_F11	Additional symptom-related items

5. Quality-of-Life Diary Data (WURSS-21)

These variables provide the **individual diary responses** underlying analyses of the WURSS-21 Quality-of-Life composite score, including AUCs, durations, and subject-level frequencies (Tables C.4.1, C.4.9, C.4.12).

Evening QoL Diary (QS3)

Variable	Description
QS3DT	Diary date
QS3DAYS	Study day number
QS3_F1–QS3_F10	WURSS-21 QoL item responses
QS3_F11, QS3_F12	Additional QoL-related fields

Composite QoL scores and derived endpoints were calculated solely from these per-day individual responses.

6. Common Cold Presence, Absence, and Study Flow

The following variables are used to classify subjects as having or not having common cold symptoms and to derive subject disposition and CONSORT flow summaries (Figure 1).

Variable	Description
SYMPTYN	Symptom check after randomisation
PE2COLDYN	Clinical confirmation of common cold
COLDYN1–COLDYN4	Cold-related symptom indicators
DS2YN, DS2NO	Randomisation status
DS4YN, DSTERM*	Study completion and termination information

7. Concomitant Medication (Diary-Captured)

These variables capture **individual diary entries** for concomitant treatments potentially affecting common cold symptoms and underlie analyses reported in Table C.4.5.1.

Variable	Description
CMTRTCAT	Medication category
CMTRT	Treatment name
CMDOSE, CMDOSU	Dose and unit
CMQUANT	Quantity
CMTM	Time of intake
CMTRTOTH, CMCMNT	Free-text descriptors

Each record represents one subject-day medication entry.

8. Global Evaluation of Efficacy and Tolerability

The following variables underlie global assessments of efficacy and tolerability reported in Tables C.4.40 and B.5.7.

Variable	Description
QS_1A, QS_2A	Global evaluation of efficacy
QS_1B, QS_2B	Global evaluation of tolerability

9. Adverse Event Data

Individual adverse event records underlying all safety summaries (e.g. Table B.5.2.1.1.1).

Variable	Description
AETERM	Adverse event term
AECAT	Event category
AESER	Serious adverse event indicator
AESTDT, AEENDT	Event start and end dates
AEONGO	Ongoing event flag
AEREL1	Relationship to investigational product
INTENSIT	Event intensity/severity

Each row corresponds to one adverse event for one subject.

10. Data Transparency Statement

The eCRF export provided with the manuscript contains **all variables listed above at individual subject- and visit/day-level**, without aggregation or transformation.
All summary statistics reported in the manuscript and in *S1_File Data in manuscript* were derived exclusively from these raw variables.
